# Sol-Gel Heterogeneization of an Ir(III) Complex for Sustainable Visible-Light Redox Photocatalysis

**DOI:** 10.3390/molecules30081680

**Published:** 2025-04-09

**Authors:** Janira Herce, Mónica Martínez-Aguirre, Javier Gómez-Benito, Miguel A. Rodríguez, Jesús R. Berenguer

**Affiliations:** Organometallic Molecular Materials (MATMO), Departamento de Química-Instituto de Investigación en Química (IQUR), Universidad de La Rioja, Madre de Dios, 53, E-26006 Logroño, La Rioja, Spain; janira.herce@unirioja.es (J.H.); m.martinez-aguirre@imperial.ac.uk (M.M.-A.); javiergobe01@gmail.com (J.G.-B.)

**Keywords:** organometallo–ionosilica, visible-light electron transfer photocatalysis, heterogeneous photocatalysis, cyclometalated iridium(III) complex

## Abstract

Photocatalysis is a key strategy for the development of sustainable solar-driven chemical processes. In this work, we report the synthesis and characterization of a novel organometallo–ionosilica material derived from the self-condensation of an alcoxysilane functionalized Ir(III) complex. In acetonitrile suspension, the material retains the photophysical properties of its precursor in solution in the same solvent, together with a significant absorption in the visible between 400 and 500 nm. As a heterogeneous photocatalyst, the material showed high efficiency in the reductive dehalogenation of 2-bromoacetophenone under blue light irradiation, achieving high yields of conversion of about 90%, and excellent recyclability in seven catalytic cycles, retaining more than 70% of the catalytic efficiency. All these properties of the self-condensed material highlight its potential as an efficient and sustainable heterogeneous photocatalyst for applications in organic synthesis and solar-driven redox processes.

## 1. Introduction

In recent decades, growing concerns about the scarcity of fossil fuels and their environmental impact have driven the search for sustainable energy sources. Among these, solar energy has emerged as a promising alternative due to its inexhaustible supply and low environmental footprint. Beyond photovoltaic electricity generation, solar energy can also drive chemical reactions through photocatalysis, a process that, inspired by natural photosynthesis, converts light energy into chemical energy, allowing reactions to occur under milder and more efficient conditions [1]. As a result, photocatalysis has become a key tool in the development of sustainable technologies for solar fuel production, environmental remediation, and the synthesis of high-value compounds [2,3,4].

The foundations of modern photochemistry were established in the late 19th century by Ciamician and Silber, pioneers in the use of light energy for organic synthesis [5]. Their founding work on photoreduction and photoisomerization remains relevant today and serves as the basis for light-driven chemical transformations [6]. Since then, photocatalysis has evolved significantly, particularly with the development of visible-light-activated systems. Visible light is less energetic than UV, reducing unwanted side reactions and making it a safer and more accessible alternative [7,8]. Recent advances in light-emitting diode (LED) technology, which provides high-intensity visible light in a controlled-wavelength range, have further expanded photocatalytic applications [9], including organic synthesis, CO_2_ reduction, and solar fuel production [10].

Therefore, efficient use of visible light, or even better solar radiation, is desirable and can be effectively achieved using organometallic and coordination complexes of transition metals [11]. Photocatalysts for visible light photocatalysis must meet basic criteria [12], including strong absorption in the visible region, long-lived excited states, and tunable redox properties to facilitate single-electron transfer (SET) or energy transfer (EnT) processes [4]. In this area, cyclometalated Ru(II), Ir(III), and Pt(II) complexes have gained prominence as visible light photocatalysts due to their high photochemical and electrochemical stability [13,14,15]. Among them, Ir(III) complexes exhibit exceptional photophysical and electronic properties, arising from strong spin-orbit coupling that allows efficient intersystem crossing transitions to long-lived triplet states. These properties, combined with their structural versatility, make them ideal candidates for a wide range of applications, including organic light-emitting diodes (OLEDs) and visible light-driven catalytic processes [16]. The ability to fine-tune their redox and photophysical properties through ligand design further enhances their versatility in synthetic and applied chemistry [17].

Despite their advantages, Ir(III) complexes have one major limitation: their use in homogeneous catalysis complicates their recovery and reuse, increasing costs and raising environmental concerns [13,14,18]. A promising strategy to overcome this problem is the heterogeneization of these complexes by using suitable inorganic substrates, such as metal nanoparticles, MOFs, zeolites, or inorganic oxides like silica [7,19,20]. This strategy has already been successfully applied to several areas of current research interest, including water splitting [21], solar fuel production [22,23], and chemical synthesis [10,24]. In this context, our groups have recently described the in situ preparation of several hybrid organometallo–silicas based on the iridium cyclometalated complex [Ir(dfppy)_2_(dasipy)]PF_6_ (dfppy = 2-(2,4)-difluorophenylpyridinyl; dasipy = N,N′-dipropyltriethoxysilane-2,2′-bipyridine-4,4′-dicarboxamide) and their application and good recyclability in the energy transfer (EnT) photoisomerization reaction of *trans*- to *cis*-stilbene under blue illumination and mild conditions [25]. Nevertheless, these materials showed low or no efficiency for single electron transfer (SET) processes, probably due to the insulating nature of silica. To overcome this issue, we have recently reported a novel material obtained by the self-condensation of the terminal alcoxysilane groups of the complex [Ir(dfppy)_2_(dasipy)]PF_6_ [26]. This material consists of discrete nanoparticles formed exclusively by the organometallic cations linked by Si–O bonds, and the anionic PF_6_^−^ counterparts. Thus, this organometallo–ionosilica has to be considered as a new type of ionosilica [27,28,29], a class of materials with potential applications in energy storage, catalysis, and biomedicine. In fact, it is highly efficient in photoredox reactions under blue light in both oxidative and reductive pathways, with excellent recyclability [26].

All these nanostructured heterogeneous photocatalysts [25,26] have been designed starting from the highly emissive derivative [Ir(dfppy)_2_(dasipy)]PF_6_. Similar to other heteroleptic cyclometallated Ir(III) complexes, in this derivative the LUMO, where the electronic charge is concentrated upon excitation of the molecule, is mainly located on the diimine ligand (dasipy, N^˄^N), which is functionalised with the terminal alkoxysilane groups. The objective of this work is therefore twofold: firstly, to achieve the steric liberation of the diimine ligand from these functional groups and, secondly, to simultaneously increase the electronic delocalisation of this ligand. The alkoxysilane groups should therefore be placed on the cyclometallated ligands (C^˄^N), which are usually less susceptible to functionalisation. In this paper we describe the synthesis of the complex [Ir(Si-dfbzapy)_2_(pyraphen)]PF_6_ (**2**), which fulfils both requirements, and its self-condensation reaction to give the new organometallo–ionosilica **SC-2**. We have also evaluated the photocatalytic activity and reusability of this material under heterogeneous conditions in a photoredox reaction. Specifically, we chose the reductive dehalogenation of 2-bromoacetophenone, as this reaction has been widely used as a benchmark for assessing photocatalyst performance under both homogeneous and heterogeneous conditions [30,31]. The reaction yields acetophenone, a non-toxic precursor in organic synthesis that is widely used in cosmetics, pharmaceuticals, and resins [32]. Through comprehensive characterisation and catalytic testing of **SC-2**, this study aims to contribute to the development of sustainable photocatalytic systems with potential applications in energy conversion and environmental sciences.

## 2. Results

### 2.1. Synthesis and Characterization of Complexes ***1*** and ***2*** and the Self-Condensed Ionosilica ***SC-2***

**Figure 1:**
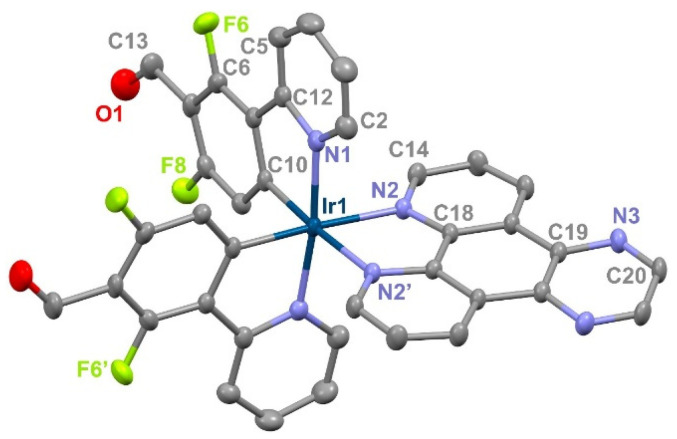
Molecular structure of the complex cation [Ir(dfbzapy)_2_(pyraphen)]^+^ (**1**^+^). Most relevant bond lengths (Å): Ir-C 2.006(4); Ir-N(1) 2.044(4); Ir-N(2) 2.141(2).

**Scheme 1:**
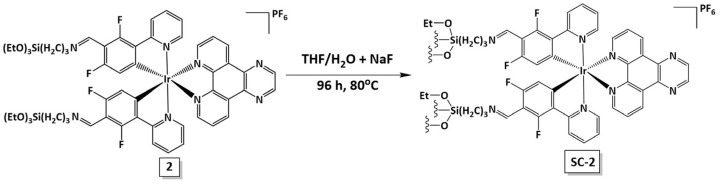
Synthesis of the organometallo–ionosilica **SC-2**, along with its proposed average composition.

With the aim of obtaining a final Ir(III) complex with cyclometalated ligands functionalized with alcoxysilane groups, the HC^^^N compound 2,6-difluoro-3-(pyridine-2-yl) benzaldehyde (dfbzapyH) [33] was used to obtain the organometallic chloride-bridged [Ir(dfbzapy)_2_(μ-Cl)]_2_ and the cationic solvate [Ir(dfbzapy)_2_(NCMe)_2_]PF_6_ precursors, following the procedure described in the bibliography [34]. The reaction of the solvate precursor with the diimine ligand pyrazino[2,3-f][1,10]phenanthroline (pyraphen) in refluxed dichloromethane for 6 h afforded the cationic complex [Ir(dfbzapy)_2_(pyraphen)]PF_6_ (**1**, see Section 3 for more details, and Scheme S1 in SI). The imination reaction of the aldehyde fragments of complex **1** with 3-aminopropyltriethoxysilane (APTES) in THF for 1 h yielded complex [Ir(Si-dfbzapy)_2_(pyraphen)]PF_6_ (**2**), which is functionalized with terminal alcoxysilane groups on both of the cyclometalated ligands (see SI, Scheme S2). Due to its high tendency to self-condense in the solid state, preventing further solubility, complex **2** was not isolated in solid state and was therefore handled in THF solution. Complexes **1** and **2** were characterized by high resolution mass spectrometry (ESI) and multinuclear ^1^H, ^13^C{^1^H} and ^19^F NMR spectroscopy. In both cases, NMR spectra show the presence of only one diimine pyraphen ligand and the equivalence of both cyclometalated groups (see Section 3, and Figures S1 and S2 in SI). In addition, complex **1** was characterized in the solid state by X-ray crystallography (Figure 1, see also Tables S1 and S2 in the SI). The molecular cation ([1]+) presents the expected pseudo-octahedral environment with the nitrogen atoms of both cyclometallated ligands in a mutually *trans* disposition, which is also the geometry expected for complex **2** and the molecules integrating the material **SC-2**. The bond distances and angles (see SI, Table S1) are similar to those observed for other related cationic heteroleptic [Ir(CN)_2_(NN)]^+^ derivatives [34]. The complex crystallizes in the centrosymmetric *C*_2/c_ space group with both enantiomers (*Δ* and *Λ*) present in the unit cell. The synthesis of the self-condensed material **SC-2** was achieved by controlled addition of the solution of complex **2** in THF over water. Stirring the mixture in the presence of NaF under reflux for 96 h afforded the ionic material **SC-2** as a yellow solid (Scheme 1, see Section 3 for more details). This material consists exclusively of cationic molecules of complex **2**, which are covalently linked through the condensation of the terminal alkoxysilane groups, together with the corresponding PF_6_^−^ anions.

**Figure 2:**
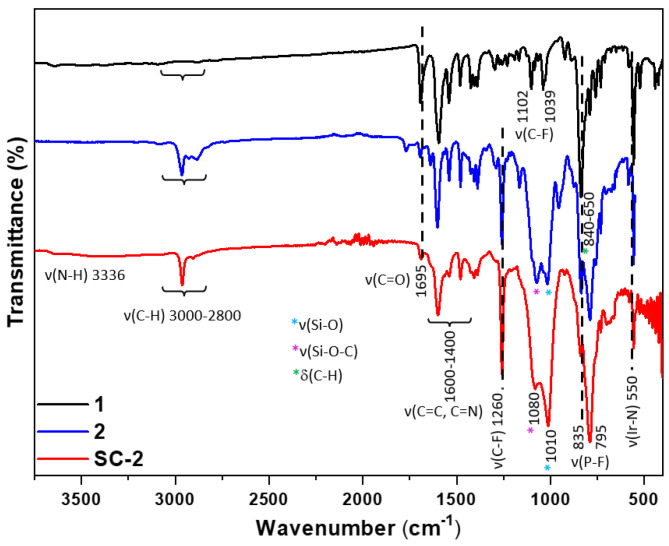
FTIR spectrum of complexes **1**, **2** and the self-condensed material **SC-2**.

Figure 2 shows the IR spectra of complexes **1**, **2** and the ionosilica **SC-2**, showing in all the cases the appearance of a P-F stretching vibration at ca. 800 cm^−1^, indicative of the presence of PF_6_^−^ anions. According to the imination process of the aldehyde groups of complex **1** (SI, Scheme S2), there are several differences between the spectra of complexes **1** and **2**. For instance, the disappearance of the ν(C=O) band at 1963 cm^−1^ observed for complex **1** and the appearance in the spectrum of complex **2** of more intense signals in the high-energy region (around 2950 cm^−1^), attributed to the C–H stretching vibrations of aliphatic groups, as well as two very strong absorptions corresponding to the stretching vibrations of Si–O–C (1071 cm^−1^) and Si–O (1006 cm^−1^) bonds. As expected, the IR spectrum of the self-condensed material **SC-2** is quite similar to that observed for complex **2**, indicating that the cyclometalated Ir(III) cations retain their structure during the condensation process. Analogous to the synthesis of a related ionosilica based on complex [Ir(dfppy)_2_(dasipy)]PF_6_, recently reported by our group [26], the main difference between the spectra of complex **2** and material **SC-2** is the significant weakness of the absorption bands related to Si–O–C bonds in the material. Since **SC-2** consists of self-condensed molecules of complex **2**, each containing six ethoxide groups susceptible to hydrolysis and condensation, it is highly probable that not all these groups undergo complete hydrolysis to form Si–O–Si bonds. Indeed, these reactions may not occur to the same extent in all molecules. Scheme 1 therefore represents an average molecular model of this material. Under these conditions, the hydrolysis of two ethoxide groups per triethoxysilane moiety is assumed, resulting in a material with the empirical formula C_48_H_42_F_10_IrN_8_O_4_Si_2_P, considering the presence of the PF_6_^−^ anion. This empirical formula agrees with the elemental analysis results, which consistently give values corresponding to the presence of eight water molecules per cationic unit (see Experimental Section in SI). Finally, the preservation of the structural integrity of the cationic organometallic fragments is in agreement with the MALDI(+) spectrum in the solid state, which shows a signal at *m*/*z* 1065 consistent with the isotopic distribution of the ionization fragment [**SC-2** − {(CH_2_)_3_Si(OEt)_3_} + 3H]^+^. It should be noted that the MALDI(−) spectrum also shows a signal at *m*/*z* 145 corresponding to the presence of the PF_6_^−^ anion (see SI, Figure S3).

**Figure 3:**
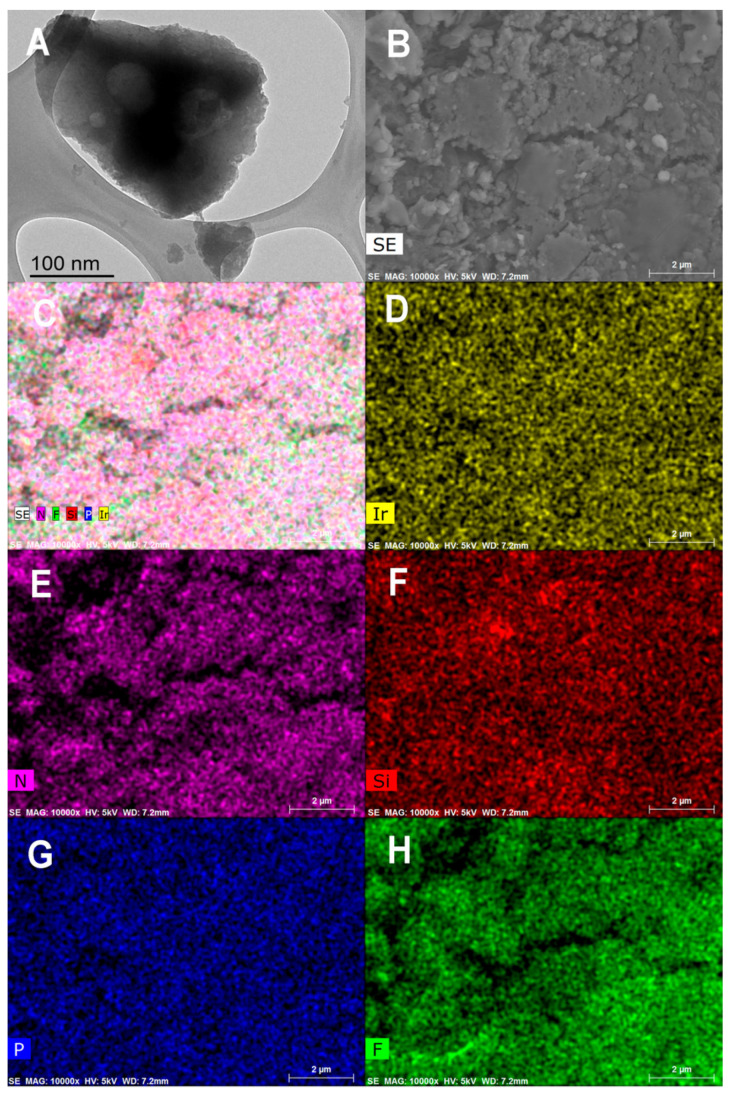
Representative TEM ((**A**), scale bar = 100 nm) and FESEM ((**B**), scale bar = 2 µm) images of the self-condensed material **SC-2**, with the corresponding layered image (**C**) combining the FESEM-EDX elemental mapping images of Ir (**D**), N (**E**), Si (**F**), P (**G**), and F (**H**) (scale bar in C–H = 2 µm).

The TEM images (Figure 3; see also Figure S4 in SI) show that **SC-2** consists of aggregates with sizes ranging between 200 and 1000 nm, formed by the agglomeration of smaller nanoparticles. The FESEM analysis also shows the granular texture typical of silica-based materials obtained by hydrolysis of alcoxysilane groups [35], while the electron diffraction pattern of **SC-2** confirms the amorphous nature of the material (see SI, Figure S4). Elemental mapping of **SC-2** using FESEM-EDX analysis (Figure 3) reveals a homogeneous distribution of atoms over the surface of the material, with a uniform composition throughout the structure. Notably, phosphorus atoms are exclusively associated with the anion PF_6_^−^, confirming their integral role in the material. These anions are likely to be located not only on the surface but also within the structure to maintain ionic neutrality.

### 2.2. Photophysical and Electronic Characterization

**Figure 4:**
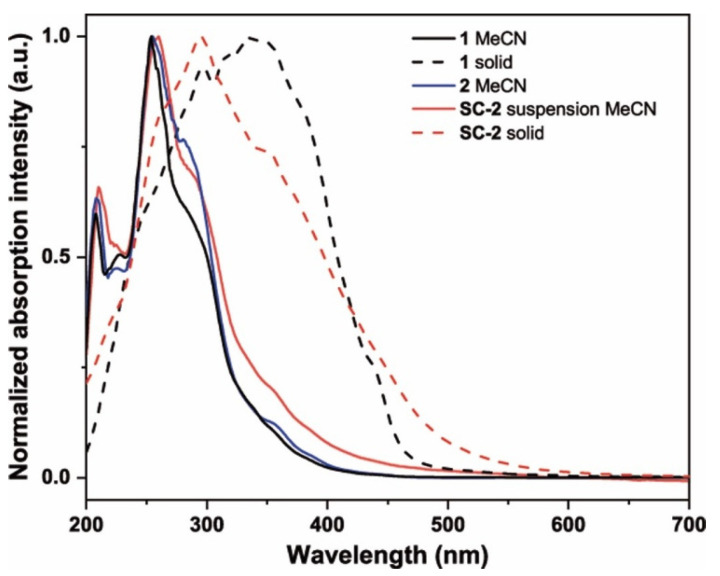
Normalized absorption spectra of complex **1** and **SC-2** in solid state, complex **1** and **2** in solution (MeCN, 5 × 10^−5^ M), and **SC-2** in suspension of MeCN (1 mg/mL).

**Figure 5:**
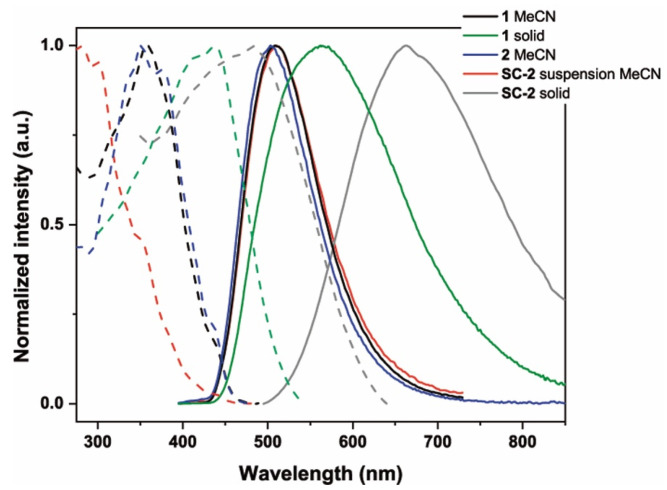
Normalized excitation (dotted line) and emission (solid line) spectra of **1** and **SC-2** in solid state, **1** and **2** in solution (MeCN, 5 × 10^−4^ M), and **SC-2** in suspension of MeCN (1 mg/mL) at room temperature.

Table 1 and Table 2 and Figure 4 and Figure 5 (see also SI, Figure S5) show the absorption and emission data of complexes **1**, **2** and material **SC-2** at room temperature. Figures S5 and S6 and Tables S3 and S4 (see SI) also present the electrochemical properties of complexes **1**, **2** and material **SC-2** at room temperature. The spectra of complex **2** has not been recorded in the solid state due to its high tendency to self-condense in that state. Solution or suspension studies were carried out in acetonitrile, as this is the solvent in which the photocatalytic studies were carried out (see below).

As observed in Figure 4, the spectra of complexes **1** and **2** in acetonitrile solution, as well as that of the **SC-2** material in suspension in the same solvent, are very similar, and have been assigned according to the spectra observed in the literature for other related cyclometalated Ir(III) derivatives [26] and the theoretical calculations carried out for complex **2** in MeCN solution (see SI, Figures S7–S9 and Tables S5–S7). Both spectra show the higher-intensity bands between 250 and 300 nm that can be mainly ascribed to spin-allowed intraligand (^1^IL or ^1^IL’; L = C^^^N, L’ = N^^^N) π-π* transitions with a certain metal contribution. Between 300 and 400 nm, the spectra show less intense allowed features related with mixture of intraligand transitions and ligand-to-ligand (^1^LL’CT; C^^^N→N^^^N) or metal-to-ligand (^1^ML’CT; Ir→N^^^N) charge transfer transitions. Finally, at lower energies (ca. 440 nm), very weak absorptions appear, more intense for the suspension of **SC-2** in MeCN, mainly related to the HOMO-LUMO gap. As observed in the theoretical calculations carried out on complex **2** in MeCN solution (see SI, Table S5 and Figure S7), the HOMO is mainly located on the iridium atom (31%) and the phenyl rings of the cyclometalated ligands (68%), while the LUMO is centred on the diimine ligand (98%). Thus, these low-energy absorptions are attributed to a mixture of ligand-to-ligand and metal-to-ligand charge transfers, traditionally ascribed to spin-forbidden excitations (^3^ML’CT/^3^LLCT), due to the strong coupling (SOC) associated with iridium [36]. As expected, the profile observed for the solid state DRUV (Diffuse Reflectance UV-vis, Figure 4) of **1** and **SC-2** appear to be quite different. Nevertheless, the spectra show similar absorption maxima to those of the solution spectra (Table 1). Finally, it should be noted that the low energy absorption observed for **SC-2** at 440 nm in the solid state display a long tail up to about 600 nm.

As observed in Figure 5 (and Table 2), the emission spectra of complexes **1** and **2** in MeCN solution, as well as the spectra of a suspension of **SC-2** in the same solvent, are similar, showing an unstructured green phosphorescence with long lifetimes (τ 12–20 μs) and low quantum yields (ϕ 1.2–7%). The SOMO and SOMO-1 orbitals calculated for complex **2** (see SI, Figure S9) show a similar composition to that described for the LUMO and HOMO (see SI, Figure S7), so this emission can be attributed to a mixture of ^3^ML’CT/^3^LL’CT excited states, with a remarkable metallic contribution. For complex **2**, the emission maximum (λ_em_ 500 nm) is slightly blue-shifted with respect to that of complex **1** (λ_em_ 510 nm), probably due to the change in the phenyl ring of the cyclometalated ligands from an aldehyde group in complex **1** to a more donor imine group in complex **2**. However, the emission maximum of the suspension of **SC-2** in MeCN is again slightly shifted to 510 nm. The solid-state spectrum of complex **1** shows a less intense phosphorescence (τ 13.24 μs, ϕ 1%) with a broader profile and a bathochromic shift (Δλ_em_ 50 nm), although this yellow emission can still be mainly attributed to the mixture of ^3^ML’CT/^3^LL’CT excited states. More surprising is the large shift observed in the spectrum of the solid **SC-2** material, which shows a low intensity and broad red phosphorescence (λ_em_ 660 nm, τ 10.73 μs, ϕ 1%). This emission could be due to heterogeneity effects of the amorphous material and/or to the formation of excimers absents in suspension due to the interaction of the molecular cations located on the surface of the material with the polar solvent. The energy of the first triplet state (T_1_) and the optical gap between the ground state (S_0_) and the T_1_ (*E*_0,0_(T_1_)) of complexes **1** and **2** (solution) and material **SC-2** (suspension) have been calculated from the excitation and emission spectra (Figure 5 and Figure S5, Table S3). In all the cases, the experimental T_1_ energy values are practically identical (ca. 2.83 eV) and agree well with that calculated theoretically by DFT for complex **2** (2.74 eV), while the suspension of **SC-2** shows the larger value of the optical gap (2.85 eV for **SC-2** vs. 2.78 eV for **1** and **2**). As a model for the electrochemical properties of the material **SC-2** in acetonitrile suspensions, the determination by cyclic voltammetry (CV) of the ground-state potential of complex **2** in MeCN solutions (*E*_ox_^1/2^ 1.72 V, *E*_red_^1/2^ −1.61 V. See SI, Figure S6 and Table S4) gives an estimated HOMO-LUMO gap of 3.33 V for this complex, which is also in agreement with the value obtained by theoretical calculations (TD-DFT 3.35 V). Finally, all these data allow the determination of the excited-state redox potential of complex **2** in MeCN (E^*^_red_ 1.17 V, E^*^_ox_ −1.06 V, Table S4) [37].

**Table 2:** Photophysical data in solid state for complex **1**, and **SC-2** in solid state, complex **1** and **2** in solution (MeCN, 5 × 10^−4^ M), and **SC-2** in suspension of MeCN (1 mg/mL). All data at room temperature.

Sample	Medium	λ_em_/nm (λ_exc_/nm)	τ/µs ^1^	Φ/%
**[Ir(dfbzapy)_2_(pirafen)]PF_6_ (1)**	Solid	560 (440)	13.24	1
	MeCN	510 (360)	12.62	7
**[Ir(Si-dfppy)_2_(pirafen)]PF_6_ (2)**	MeCN	500 (375)	20.19	1.2
**SC-2**	Solid	660 (475)	10.73	1
	Suspension MeCN	510 (360)	13.79	2.4

^1^ Emissions lifetimes calculated as mono-exponential decay or an average of a bi-exponential decay.

### 2.3. Photocatalytic Studies

**Scheme 2:**
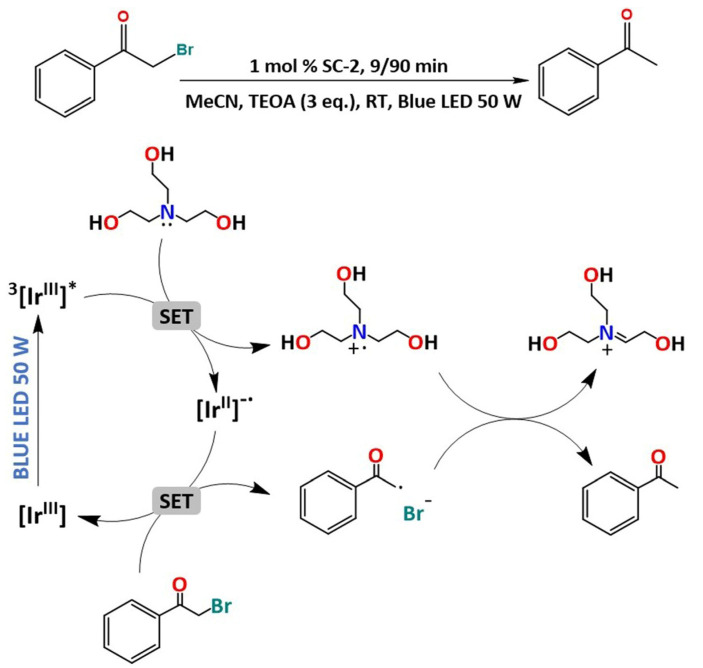
Photocatalytic dehalogenation of 2-bromoacetophenone (**up**) and mechanism of the reaction (**down**).

As the organometallo–ionosilica **SC-2** exhibits absorption and excitation maxima at λ_abs_ > 400 nm in acetonitrile suspensions (Figure 4 and Figure 5), its photocatalytic activity has been evaluated under blue light illumination (50 W blue LED, λ_max_ = 450 nm). The reductive dehalogenation reaction of 2-bromoacetophenone was chosen for this purpose (Scheme 2, up). This single electron transfer (SET) reaction requires the presence of three equivalents of triethanolamine (TEOA) as a sacrificial reagent. The reaction starts with the excitation of the photocatalyst, resulting in a spin-allowed metal-to-ligand charge transfer (^1^MLCT), followed by intersystem crossing from the singlet excited state (^1^MLCT) to a lower-energy triplet state (^3^MLCT). The excited iridium species reacts with triethanolamine, producing a reduced iridium radical and a radical cation derived from triethanolamine. The iridium radical anion then returns to its ground state by transferring an electron to 2-bromoacetophenone, thereby facilitating the cleavage of the C-Br bond. This process results in the formation of a bromide anion and an acetophenone radical. The latter abstracts a hydrogen atom from the triethanolamine radical cation, yielding an iminium cation and acetophenone (Scheme 2, down). The reaction progress was monitored by ¹H NMR spectroscopy, using 1,3,5-trimethoxybenzene as an internal standard (see SI, Figure S10). Initially, the reaction with the **SC-2** photocatalyst was carried out under N_2_ atmosphere on an NMR scale, using deuterated acetonitrile as the solvent, starting from 15 mg of 2-bromoacetophenone and a catalyst loading of 1 mol% (Table 3). Under these conditions, the reaction gives about 90% yield of acetophenone within 9 minutes, but with the complete consumption of the starting bromoacetophenone. This makes **SC-2** a more efficient photocatalyst than the related iridium-based self-condensed material recently reported by us [26], which achieves a similar 90% yield but in 90 min under the same conditions. In addition, the complete consumption of bromoacetophenone indicates the occurrence of a secondary reaction causing the decomposition of approximately 10% of the starting material. Notably, no additional aromatic signals were observed in the final NMR spectra. This behaviour could be explained by the greater electronegativity of bromine compared to carbon, which withdraws electron density from the C-Br bond, thereby increasing the positive charge density of the carbonyl group. As a result, 2-bromoacetophenone would become more reactive towards nucleophilic species present in the reaction medium, starting the decomposition reaction. Also, in the absence of blue light irradiation or TEOA, no reaction was observed in the control reactions (Table 3).

To evaluate the recyclability of the heterogeneous photocatalyst and to facilitate its recovery, the process was scaled up while maintaining the same light source under N_2_ atmosphere. The first cycle was started with 36.5 mg of **SC-2** (1 mol% catalyst loading), using 0.65 g of 2-bromoacetophenone, 0.30 g of trimethoxybenzene, and 1.3 mL of triethanolamine in each cycle. After each cycle, the photocatalyst was recovered by centrifugation. The material was first washed with water to remove the iminium salt formed during the reaction. It was then washed with absolute ethanol and acetonitrile before being dried and reused in the next catalytic cycle (see Section 3 for more details).

**Figure 6:**
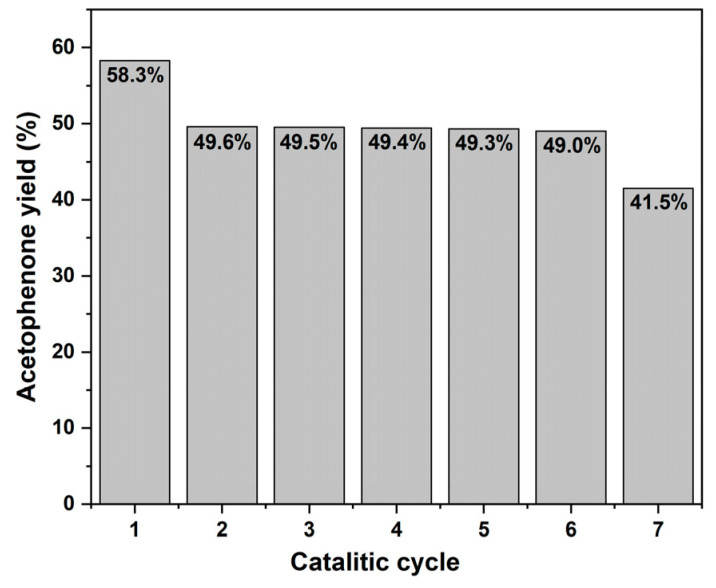
Results of the conversion values from the recovery and reuse of **SC-2** over seven consecutive catalytic cycles, each lasting 90 min. All experiments were conducted under identical conditions.

As can be seen in Figure 6, under these conditions, the reaction of the first cycle reaches approximately 60% conversion after 90 min, while the maximum conversion obtained in the last cycle, in the same time period, is 41.5%. This indicates a loss of the catalytic capacity of the **SC-2** material of less than 30% over the seven cycles recorded. Furthermore, two significant decreases were observed (about 9% after the first and sixth cycles), while the performance remained practically stable in the rest of the cycles. This suggests that some photocatalyst may have been lost during the washing processes. This finding is consistent with the observation that no signals corresponding or compatible with the presence of compound **2** have been detected by RMN spectroscopy in the reaction medium of any of the cycles, suggesting that no significant leaching has occurred during the reaction.

Finally, to further study the possible photodegradation of **SC-2**, the material used in up to seven catalytic cycles was recovered and studied by means of infrared, absorption, and emission spectroscopies (see SI, Figures S11–S13 and Tables S8 and S9). The IR spectrum of the reused **SC-2** material (Figure S11) is quite similar to that of the original material, with the most significant profile modification occurring in the region corresponding to the absorptions derived from the aromatic groups (1300–1600 cm^−1^). The DRUV spectra of both materials (Figure S12, Table S8) shows the largest changes. The absorptions at lower energies (λ_abs_ 350–450 nm; ML’CT/LL’CT) are less intense in the spectrum of the reused material, although they appear at similar wavelengths than those observed for the original material. However, the profile of the absorptions between 250 and 300 nm shows a greater change with an increase in the intensity of the bands at ca. 260 nm. These modifications in the high-energy bands have previously been associated with structural changes in the aromatic ligands [37], which would be consistent with the feature observed in the infrared spectrum, indicating some photodegradation of the molecular cations located onto the surface of the material. However, despite the aforementioned, the emission spectra of both materials (Figure S13, Table S9) show similar unstructured red phosporescence at 660 nm, with a small decrease in lifetime (10.7 vs. 13.7 μs), indicating that the bulk of the material exhibits adequate photostability after seven cycles of use.

## 3. Materials and Methods

### 3.1. General Methods

The compound 2,6-difluoro-3-(pyridine-2-yl) benzaldehyde (dfbzapyH) [33] and the precursors [Ir(dfbzapy)_2_(μ-Cl)]_2_ and [Ir(dfbzapy)_2_(NCMe)_2_]PF_6_ were synthesized by procedures described in the bibliography [34]. The ligand pyraphen = pyrazino[2,3-f][1,10]phenanthroline is commercially available. All synthetic procedures for complexes 1 and 2 were performed under an inert atmosphere and in anhydrous conditions. Commercially sourced reagents were used as received, without additional purification. Solvents were obtained from a solvent purification system (M-BRAUN MS SPS-800). Elemental analyses of the compounds were recorded on a Thermo Finnigan Flash 1112 microanalyzer (Thermo Fischer Scientific, Waltham, MA, USA). Mass spectra were made on a Microflex MALDI-TOF Bruker (MALDI) (Bruker, Billerica, MA, USA) spectrometer, operating in the linear and reflector modes using dithranol as matrix, and on a Bruker Microtof-Q spectrophotometer (Bruker, Billerica, MA, USA) with ESI/APCI interface in positive ion mode, using a 90:10 methanol:water mixture and 0.1% formic acid as mobile phase. NMR spectra of the compounds were obtained on Bruker ARX-300 and ARX-400 spectrometers (Bruker, Billerica, MA, USA) using CH_3_CN and acetone-d_6_ as solvent. Chemical shifts are presented in parts per million (ppm) relative to external standards (SiMe_4_ for ^1^H and ^13^C{^1^H} and CFCl_3_ for ^19^F{^1^H}) and coupling constants in hertz (Hz). ^1^H-^1^H COSY, ^1^H-^13^C HMBC and ^1^H-^13^C HSQC correlation experiments have been used for the assignation of the ^1^H and ^13^C{^1^H} NMR spectra. IR spectra were recorded on a Perkin Elmer FT-IR Spectrometer Spectrum Two in the wavenumber range from 4000 to 400 cm^−1^. The UV-VIS absorption spectra in solution were obtained using a Hewlett Packard 8453 spectrophotometer (Agilent Technologies, Santa Clara, CA, USA). Solid-state Diffuse Reflectance UV-VIS (DRUV) spectra were recorded on a Shimazdu UV-3600 spectrophotometer (Shimadzu Corporation, Kyoto, Japan) with a Harrick Praying Mantis accessory and recalculated using the Kubelka Munk function. The samples were diluted in KBr powder for the complex and silica for the materials. Excitation and emission spectra were measured on an Edinburgh FLS 1000 spectrofluorimeter (Edinburg Instruments Ltd., Edinburg, UK), where lifetime measurements were also performed with a µF2 pulse lamp (Power: 100 W, Fuse: 3.15 Amp A/S). Quantum yields were measured with Hamamatsu Absolute PL Quantum Yield Measurement System (Hamamatsu Photonics, Shizuoka, Japan). Cyclic voltammetry and differential pulse voltammetry measurements were carried out on a Voltalab PST050 electrochemical workstation (Volta Labs, Boston, MA, USA), using a 0.1 M solution of tetrabutylammonium hexafluorophosphate (NBu_4_PF_6_) as the supporting electrolyte. A conventional three-electrode configuration was used, consisting of a platinum spar working electrode, a platinum wire counter electrode, and an Ag/AgCl reference electrode. As the solvent, was used CH3CN ([1] 10^−4^ M) under inert atmosphere and the ferrocene/ferrocenium couple served as internal reference.

The morphology of the materials was investigated by transmission electron microscopy (TEM) and Scanning Electron Microscopy (SEM). Samples were prepared by dipping a sonicated suspension of the sample in ethanol on a carbon-coated copper. TEM images were performed using a JEM-2010 microscope (JEOL, 200 kV, 0.14 nm of resolution) (JEOL Ltd., Tokyo, Japan), with a detector of Si(Li) (area of 30 mm^2^ and resolution of 142 eV). The digital analysis of the TEM micrographs was performed using DigitalMicrographTM 3.6.1. by Gatan. SEM analyses were carried out in a field emission scanning electron microscope (FESEM) Merlin VP Compact (Zeiss, 1.6 nm of resolution at 1 kV) (Zeiss, Wetzlar, Germany), where EDX analyses were also performed.

### 3.2. Synthesis of [Ir(Dfbzapy)_2_(pyraphen)]PF_6_ (***1***)

0.83 g (0.36 mmol) of pyrazino[2,3-f][1,10]phenanthroline (pyraphen) were added to a solution of 0.37 g (0.46 mmol) of [Ir(dfbzapy)_2_(NCMe)_2_]PF_6_ in 25 mL of dichloromethane, and the mixture was refluxed for 6 h in the absence of light. After the reaction time, the mixture was evaporated to dryness, and the solid residue was treated with diethyl ether and hexane to yield complex **1** as a yellow solid (0.35 g, 81%). Anal. Calc. for C_38_H_20_F_10_IrN_6_O_2_P: C, 45.38; H, 2.00; N, 8.36. Found: C, 45.38; H, 2.21; N, 9.70.ESI (+): *m*/*z* 861 [Ir(dfbzapy)_2_(pyraphen)]^+^ (100%). IR (cm^−1^): ν(C-H_aromatic_) 3096 (w); ν(C=O) 1693 (m); ν(C=C, C=N) 1595 (s), ν(C-F) 1102 (m), 1039 (s); ν(P-F) 837 (vs); ν(Ir-N) 557 (s). ^1^H NMR (400 MHz, acetone-d_6_, δ): 10.30 (s, 2H, CHO); 9.83 (dd, J_H-H_ = 9.0, 1.6 Hz, 2H, H^2′^); 9.36, (s, 2H, H^8′^); 8.80 (dd, J_H-H_ = 5.4, 1.6 Hz, 2H, H^4′^); 8.51 (d, J_H-H_ = 8.4 Hz, 2H, H^2^); 8.25 (dd, J_H-H_ = 8.5, 5.2 Hz, 2H, H^3′^); 8.11 (t, J_H-H_ = 7.9, 2H, H^3^); 7.96 (d, J_H-H_ = 5.0 Hz, 2H, H^5^); 7.16 (t, J_H-H_ = 6.1 Hz, 2H, H^4^); 6.11 (d, ^3^J_H-F_ = 10.3 Hz, 2H, H^9^). ^13^C{^1^H} NMR (100.6 MHz, acetone-d_6_, δ): 184.4 (t, ^3^J_C-F_ = 5.1 Hz, CHO); 164.4 (d, ^3^J_C-F_ = 8.4 Hz, C^10^); 164.2 (dd, ^1^J_C-F_ = 235.4 Hz, ^3^J_C-F_ = 6.3 Hz, C^8^); 163.8 (d, J_C-F_ = 6.6 Hz, C^11^ or C^12^); 161.5 (dd, ^1^J_C-F_ = 240.4 Hz, ^3^J_C-F_ = 5.8 Hz, C^6^); 154.2 (s, C^4′^); 151.6 (s, C^5^); 149.3 (s, C^9′^); 148.1 (s, C^8^); 141.4 (s, C^3^); 140.7 (s, C^6′^); 137.1 (s, C^2′^); 132.1 (s, C^5′^); 130.3 (s, C^11^ or C^12^); 129.6 (s, C^3′^); 126.0 (s, C^4^); 125.5 (d, ^5^J_C-F_ = 21.1 Hz, C^2^); 116.2 (d, ^2^J_C-F_ = 17.1 Hz, C^9^); 111.2 (pst, ^2^J_C-F_ = 12.0 Hz, C^7^). ^19^F {^1^H} NMR (376.5 MHz, acetone-d_6_, δ): −72.63 (d, J_F-P_ = 707.7 Hz, 6F, PF_6_); −114.23 (d, ^3^J_F-F_ = 7.6 Hz, 2F, F^6^); −117.43 (d, ^3^J_F-F_ = 7.6 Hz, 2F, F^8^).

### 3.3. Synthesis of [Ir(Si-Dfbzapy)_2_(pyraphen)]PF_6_ (***2***)

A solution of 0.10 g (0.09 mmol) of [Ir(dfbzapy)_2_(pyraphen)]PF_6_ (**1**) in 10 mL of THF was treated with 30.6 µL (0.20 mmol) of 3-aminopropyltriethoxysilane (APTES) in the presence of activated molecular sieves (used to collect the water produced during the reaction). The mixture was stirred for 1 hour at room temperature. The resulting yellow solution was filtered under an inert atmosphere and concentrated to ca. 3 mL. Due to the self-condensation tendency of complex **2** in the solid state, even under inert conditions, the compound cannot be isolated as a solid. Instead, the reaction mixture is directly used in the next step without further purification. The yield could not be determined. For the purposes of the subsequent reaction, a theoretical imination yield of 100% was assumed. ESI(+): *m*/*z* 1062 [{Ir(Si-dfbzapy)_2_(pyraphen)} − 4Et − {2(-OEt)]^+^ (100%). IR (cm^−1^): ν(C-H aromatic) 2947 (m); ν(C=C, C=N) 1613 (s); ν(C-F) 1260; ν(Si–O–C) 1071 (vs); ν(Si–O) 1006 (vs); ν(P-F) 836 (vs); ν(Ir-N) 570 (s). ^1^H NMR (300 MHz, acetone-d_6_, δ): 9.83 (dd, J_H-H_ = 8.3, 1.5 Hz, 2H, H^2′^); 9.37 (s, 2H, H^8′^); 8.81 (dd, J_H-H_ = 5.2, 1.5 Hz, 2H, H^4′^); 8.47 (m, 4H, N=CH y H^2^); 8.27 (dd, J_H-H_ = 8.4, 5.2 Hz, 2H, H^3′^); 8.04 (t, J_H-H_ = 7.8 Hz, 2H, H^3^); 7.92 (d, J_H-H_ = 6.1 Hz, 2H, H^5^); 7.10 (pst, J_H-H_ = 6.1 Hz, 2H, H^4^); 5.99 (d, ^3^J_H-F_ = 10.2, 2H, H^9^); 3.81 (q, J_H-H_ = 6.2 Hz, 12H, OC*H_2_*CH_3_); 3.63 (t, J_H-H_ = 6.2 Hz, 2H, N–CH_2_–); 3.16 (t, J_H-H_ = 6.6 Hz, 2H, N–CH_2_–); 1.83 (m, 2H, –CH_2_–); 1.78 (m, 2H, –CH_2_–); 1.19 (t, J_H-H_ = 6.2 Hz, 18H, OCH_2_C*H_3_*); 0.66 (m, 4H, SiCH_2_–). ^19^F NMR (282 MHz, Acetone-d_6_, δ): −72.60 (d, J_F-P_ = 707.7 Hz, 6F, PF_6_); −111.52 (d, ^3^J_F-F_ = 9.1 Hz, 2F, F^6^); −114.33 (d, ^3^J_F-F_ = 9.2 Hz, 2F, F^8^).

### 3.4. Synthesis of the Self-Condensed Material (***SC-2***)

As a continuation of the procedure for the preparation of [Ir(Si-dfppy)_2_(pyraphen)]PF_6_ (**2**) described above, the obtained solution of compound **2** in 3 mL of THF (approximately 0.09 mmol) was added dropwise to 13 mL of distilled water using an automated dropping system at a controlled rate of 0.02 mL/s to ensure thorough homogenization. After stirring at room temperature for 1 hour, 0.07 mL of a 0.05 M sodium fluoride solution was added, and the mixture was heated to 80 °C for 4 days. Upon completion of the reaction, the **SC-2** material precipitated as a yellow solid, which was isolated by centrifugation at 18,000 rpm for 30 min. The solid was subsequently washed with distilled water, ethanol, and acetonitrile, with centrifugation performed after each washing step. The washes with ethanol and acetonitrile effectively remove any unreacted complex **2**, resulting in the pure self-condensed material. (0.03 g, 30%). Anal. Calc. for C_48_H_56_F_10_IrN_8_O_4_Si_2_P: C, 45.60; H, 3.35; N, 8.86. Best analyses found: C, 39.20; H, 4.04; N, 5.22. (The self-condensed material is obtained in an aqueous medium. All the elemental analyses performed show similar values, maybe due to the presence of water and/or THF occluded in the material. In fact, the analyses were compatible with **SC-2**·8H_2_O: C, 39.10; 3.58; N, 8.29). IR (cm^−1^): ν(C-H_alifatic_) 2963 (m); ν(C=C, C=N) 1600 (s); ν(C-F) 1259 (s); ν(Si–O–C) 1082 (vs); ν(Si–O) 1013 (vs); ν(P-F) 790 (vs); ν(Ir-N) 557 (s). MALDI (+): *m*/*z* 1065 [**SC-2** − {(CH_2_)_3_Si(OEt)_3_} + 3H]^+^ (1%); 861 [**SC-2** − {(CH_2_)_3_Si(OEt)_3_} − (pyraphen) + Et + 2H] + (100%). MALDI (−): *m*/*z* 145 [PF_6_]^-^ (100%).

### 3.5. X-Ray Crystallographic Analysis of Complex ***1***

Selected bond lengths and angles and X-ray crystallographic data for complex **1**·1.5(CH_3_)_2_CO are summarized in SI (Tables S1 and S2). Orange crystals of complex **1** were obtained by slow diffusion at room temperature of *n*-hexane into a saturated solution of acetone. X-ray intensity data were collected with a Bruker D8 QUEST (PHOTON 100 CMOS) (Bruker, Karlsruhe, Germany) area-detector diffractometer, using graphite-monochromatic Mo-K_α_ radiation. The images were collected and processed using Bruker APEX4 and SAINT programs [38], carrying out the absorption correction at this point by semi-empirical methods using SADABS [38]. The structure was solved by intrinsic phasing using SHELXT [39], and refined by full-matrix least squares on F^2^ with SHELXL [40], using the WINGX program suite [41]. All non-hydrogen atoms were assigned anisotropic displacement parameters. The inspection of the structure with PLATON [42] and SQUEEZE [43] revealed the presence of two voids of 310 e·Å^−3^ in the unit cell, each of them containing 99 electrons, which fits well for the presence of a total of six acetone molecules in the unit cell (one and half acetone molecule for each iridium(III) complex molecule—**1**·1.5(CH_3_)_2_CO). Supplementary crystallographic data for 1 were deposited at The Cambridge Crystallographic Data Centre (CCDC) under deposition number CCDC 2428185. These data can be obtained free of charge via www.ccdc.cam.ac.uk/data_request/cif (accessed on 4 April 2025).

### 3.6. Theoretical Calculations

Calculations for complex **2** (MeCN) were performed with the Gaussian 16 package [44], using the Becke three-parameter functional combined with the Lee–Yang–Parr correlation functional (B3LYP) [45] with the Becke–Johnson D3BJ correction [46]. No negative frequency was found in the vibrational frequency analysis of the final equilibrium geometries. The basis set used was the LanL2DZ effective core potential for Ir and 6-31G(d,p) for the ligand atoms [47]. DFT and TD-DFT calculations were performed using the polarized continuum model approach (PCM) [48], implemented in the Gaussian 16 software, in presence of the corresponding solvents. The predicted emission wavelengths were obtained from the energy difference between the triplet state at its optimized geometry and the singlet state in the triplet geometry. The results were visualized using GaussView 6. Overlap populations between molecular fragments were calculated using the GaussSum 3.0 software [49]. The emission energy was calculated as the difference of the optimized T_1_ geometry for both states (adiabatic electronic transition).

### 3.7. Photocatalytic Methods for Dehalogenation of 2-Bromoacetophenone

Two different approaches were employed to study the photocatalytic reaction, based on the methodology previously described [26]:

*In an NMR tube:* A solution was prepared by dissolving 2-bromoacetophenone (15 mg, 0.075 mmol), triethanolamine (30 μL, 0.23 mmol), and 0.84 mg (0.75 μmol) of the photocatalyst **SC-2** in 0.8 ml of acetonitrile-d_3_. The tube was degassed under nitrogen by bubbling for 5 min in the dark, followed by sonication for 1 min. The reaction was then irradiated with a 50 W blue LED (λ_max_ = 450 nm) for 9 min while stirring. The progress of the reaction was monitored by ^1^H-NMR, with 1,3,5-trimethoxybenzene (0.06 mmol) as the internal standard, to determine the yield.

*In a round bottom flask:* Under a nitrogen atmosphere, 2-bromoacetophenone (0.65 g, 3.27 mmol), **SC-2** (36.51 mg, 0.03 mmol), and 15 mL of anhydrous acetonitrile (previously degassed by nitrogen bubbling for 20 min) were added to a 50 mL round-bottom flask. After sonicating the mixture for 1 min, triethanolamine (1.3 mL, 9.8 mmol) and 1,3,5-trimethoxybenzene (300 mg, 1.78 mmol) were added. The reaction mixture was irradiated with a 50 W blue LED (λ_max_ = 450 nm) for 90 min while being stirred, and the progress of the reaction was also monitored by ^1^H-NMR.

For the recovery and reusing of the catalyst **SC-2**, the mixture was centrifuged at 18,000 rpm for 30 min and the resulting solid was washed with distilled water and sonicated for 10 min, followed by centrifugation at 18,000 rpm for another 30 min. This washing and centrifugation process was repeated using absolute ethanol and then acetonitrile. The solid was subsequently suspended in dichloromethane, transferred back to the reaction flask, and dried under vacuum at 100 °C to remove any residual moisture. The same procedure was applied to subsequent reactions. Yields were determined using ^1^H-NMR analysis with 1,3,5-trimethoxybenzene serving as the internal standard.

## 4. Conclusions

In this study, we describe the synthesis and characterisation of a new photocatalytic material based on the Ir(III) complex [Ir(Si-dfbzapy)_2_(pyraphen)]PF_6_ (**2**), functionalized with alkoxysilane groups in the cyclometalated ligands. This complex undergoes self-condensation, leading to the formation of the organometallo–ionosilica **SC-2**. Structural and spectroscopic analyses confirm the preservation of the structure of the cationic Ir(III) complex after the heterogenization process, as well as the homogeneous distribution of the PF_6_^−^ counteranion throughout the material, highlighting its ionic nature.

From a photophysical point of view, while **SC-2** exhibits a weak red emission in the solid state, its absorption and emission spectra in acetonitrile suspension are practically identical to those of its precursor **2** in solution in the same solvent, showing a blue greenish phosphorescence and a significant absorption between 400 and 500 nanometers. In addition, the electrochemical properties calculated for **SC-2** and complex **2**, with the latter acting as a model of the material in solution, indicate its feasibility for photoinduced electron transfer processes. Thus, the **SC-2** material has shown excellent photocatalytic activity in heterogeneous conditions in the reductive dehalogenation reaction of 2-bromoacetophenone under blue light illumination (450 nm). In terms of reusability, **SC-2** retains its photocatalytic activity over seven catalytic cycles, with a decrease in efficiency under 30% and likely due to material loss during the washing steps. Nevertheless, post-reaction analysis of the material suggests the possibility of some photodegradation of surface-bound molecules during irradiation. Despite this, the material exhibits remarkable photostability, allowing it to be used in multiple catalytic cycles.

Overall, these findings highlight the potential of **SC-2** as an efficient, sustainable, and reusable heterogeneous photocatalyst for applications in organic synthesis and solar-driven redox transformations.

## Figures and Tables

**Table 1:** Absorption data of complexes **1**, **2** and self-condensed material **SC-2**.

Sample	Medium	λ_abs_/nm (ε·10^−3^/M^−1^·cm^−1^)
**[Ir(dfbzapy)_2_(pyraphen)]PF_6_ (1)**	Solid	245, 290, 341, 380, 440
	MeCN 5 × 10^−5^ M	255 (20.0), 296 (11.0), 355 (2.2), 385 (0.9), 442 (0.1)
**[Ir(Si-dfppy)_2_(pyraphen)]PF_6_ (2)**	MeCN 5 × 10^−5^ M	256 (20.0), 283 (15.4), 360 (2.3), 390 (0.9), 442 (0.1)
**SC-2**	Solid	256, 295, 350, 375, 420, 445

**Table 3:** Results of the dehalogenation reaction of 2-bromoacetophenone in heterogeneous conditions under N_2_, using the organometallo–ionosilica **SC-2** (1 mol% [Ir]) as photocatalyst.

Entry	Time (min)	Yield (%)
1	9	93 (±4) ^1^
2 ^2^	20	-
3 ^3^	20	-

^1^ Average value of three runs. ^2^ No TEOA. ^3^ No light.

## Data Availability

Data will be made available on request.

## Supplementary Materials

The following supporting information can be downloaded at: https://www.mdpi.com/article/10.3390/molecules30081680/s1. Supplementary Materials includes characterization data for complexes **1** and **2**, and material **SC-2**, including the photophysical and electronic properties, the theoretical calculations performed on complex **2**, and the photostability studies of **SC-2**.

## References

[1] Kou J., Lu C., Wang J., Chen Y., Xu Z., Varma R.S. (2017). Selectivity Enhancement in Heterogeneous Photocatalytic Transformations. Chem. Rev..

[2] Ham R., Nielsen C.J., Pullen S., Reek J.N.H. (2023). Supramolecular Coordination Cages for Artificial Photosynthesis and Synthetic Photocatalysis. Chem. Rev..

[3] Chandra P., Choudhary N., Mobin S.M. (2023). The game between molecular photoredox catalysis and hydrogen: The golden age of hydrogen budge. Mol. Catal..

[4] Millward F., Zysman-Colman E. (2023). Alchemy reimagined: Photocatalysis using anthropogenic waste materials. Trends Chem..

[5] Ciamician G., Silber P. (1908). Chemische Lichtwirkungen. Ber. Dtsch. Chem. Ges..

[6] Heindel N.D., Pfau M.A. (1965). A profitable partnership: Giacomo Ciamician and Paul Silber. J. Chem. Educ..

[7] Yakushev A.A., Abel A.S., Averin A.D., Beletskaya I.P., Cheprakov A.V., Ziankou I.S., Bonneviot L., Bessmertnykh-Lemeune A. (2022). Visible-light photocatalysis promoted by solid- and liquid-phase immobilized transition metal complexes in organic synthesis. Coord. Chem. Rev..

[8] Marzo L., Pagire S.K., Reiser O., König B. (2018). Visible-Light Photocatalysis: Does It Make a Difference in Organic Synthesis?. Angew. Chem. Int. Ed..

[9] König B. (2017). Photocatalysis in Organic Synthesis—Past, Present, and Future. Eur. J. Org. Chem..

[10] Lindroth R., Materna K.L., Hammarström L., Wallentin C.-J. (2022). Sustainable Ir-Photoredox Catalysis by Means of Heterogenization. ACS Org. Inorg. Au.

[11] Chan A.Y., Perry I.B., Bissonnette N.B., Buksh B.F., Edwards G.A., Frye L.I., Garry O.L., Lavagnino M.N., Li B.X., Liang Y. (2022). Metallaphotoredox: The Merger of Photoredox and Transition Metal Catalysis. Chem. Rev..

[12] Prier C.K., Rankic D.A., MacMillan D.W.C. (2013). Visible Light Photoredox Catalysis with Transition Metal Complexes: Applications in Organic Synthesis. Chem. Rev..

[13] Bevernaegie R., Wehlin S.A.M., Elias B., Troian-Gautier L. (2021). A Roadmap Towards Visible Light Mediated Electron Transfer Chemistry with Iridium(III) Complexes. ChemPhotoChem.

[14] Bell J.D., Murphy J.A. (2021). Recent advances in visible light-activated radical coupling reactions triggered by (i) ruthenium, (ii) iridium and (iii) organic photoredox agents. Chem. Soc. Rev..

[15] Glaser F., Wenger O.S. (2020). Recent progress in the development of transition-metal based photoredox catalysts. Coord. Chem. Rev..

[16] Singh A., Teegardin K., Kelly M., Prasad K.S., Krishnan S., Weaver J.D. (2015). Facile synthesis and complete characterization of homoleptic and heteroleptic cyclometalated Iridium(III) complexes for photocatalysis. J. Organomet. Chem..

[17] Tritton D.N., Tang F.-K., Bodedla G.B., Lee F.-W., Kwan C.-S., Leung K.C.-F., Zhu X., Wong W.-Y. (2022). Development and advancement of iridium(III)-based complexes for photocatalytic hydrogen evolution. Coord. Chem. Rev..

[18] Sakai K., Ozawa H. (2007). Homogeneous catalysis of platinum(II) complexes in photochemical hydrogen production from water. Coord. Chem. Rev..

[19] Shi J., Su Z., Li X., Feng J., Men C. (2023). Impacts of host–guest assembly on the photophysical and photocatalytic properties of heterogenized molecular photosensitizer and catalysts. J. Mater. Chem. A.

[20] Kunzmann A., Valero S., Sepúlveda Á.E., Rico-Santacruz M., Lalinde E., Berenguer J.R., García-Martínez J., Guldi D.M., Serrano E., Costa R.D. (2018). Hybrid Dye-Titania Nanoparticles for Superior Low-Temperature Dye-Sensitized Solar Cells. Adv. Energy Mater..

[21] Wang Z., Li C., Domen K. (2019). Recent developments in heterogeneous photocatalysts for solar-driven overall water splitting. Chem. Soc. Rev..

[22] Luo T., Gilmanova L., Kaskel S. (2023). Advances of MOFs and COFs for photocatalytic CO_2_ reduction, H_2_ evolution and organic redox transformations. Coord. Chem. Rev..

[23] Li Y.-L., Li A.-J., Huang S.-L., Vittal J.J., Yang G.-Y. (2023). Polypyridyl Ru(ii) or cyclometalated Ir(iii) functionalized architectures for photocatalysis. Chem. Soc. Rev..

[24] Hao Y., Lu Y.-L., Jiao Z., Su C.-Y. (2024). Photocatalysis Meets Confinement: An Emerging Opportunity for Photoinduced Organic Transformations. Angew. Chem. Int. Ed..

[25] Martínez-Aguirre M., Serrano E., Ezquerro C., Lalinde E., Berenguer J.R., García-Martínez J., Rodríguez M.A. (2023). Hybrid organometallo-silica catalysts for sustainable visible-light promoted olefin isomerization. Catal. Today.

[26] Martínez-Aguirre M., Herce J., Serrano E., García-Martínez J., Rodríguez M.A., Berenguer J.R. (2025). Self-condensed organometallo Ir(III) ionosilica for sustainable visible-light promoted electron-transfer photocatalysis. J. Catal..

[27] Tran Duy T., Duarte Rodrigues A., Vo-Thanh G., Hesemann P. (2020). Dialkyl imidazolium acetate ionosilica as efficient and recyclable organocatalyst for cyanosilylation reactions of ketones. Green Energy Environ..

[28] Wu H., Hesemann P., Trens P., Silly G., Salles F., Zajac J. (2020). Ionosilica-based anion exchangers for low-temperature thermochemical storage of energy under mild conditions of adsorbent regeneration and saturation. Chem. Eng. J..

[29] Hesemann P. (2018). Applications of ionosilicas in heterogeneous catalysis: Opportunities for the elaboration of new functional catalytic phases. Curr. Opin. Green Sustain. Chem..

[30] Materna K.L., Hammarström L. (2021). Photoredox Catalysis Using Heterogenized Iridium Complexes. Chem. Eur. J..

[31] Zhang W., Wang D., Xie Q., Xu C., Kuang G., Tang J., Pan C., Yu G. (2023). Enhanced Reducibility via Altering Exciton Binding Energy of Conjugated Microporous Polymers for Photocatalytic Reduction. Macromolecules.

[32] Devika S., Palanichamy M., Murugesan V. (2011). Selective oxidation of ethylbenzene over CeAlPO-5. Appl. Catal. A.

[33] Okamura N., Nakamura T., Yagi S., Maeda T., Nakazumi H., Fujiwara H., Koseki S. (2016). Novel bis- and tris-cyclometalated iridium (iii) complexes bearing a benzoyl group on each fluorinated 2-phenylpyridinate ligand aimed at development of blue phosphorescent materials for OLED. RSC Adv..

[34] Millán G., Nieddu M., López I.P., Ezquerro C., Berenguer J.R., Larráyoz I.M., Pichel J.G., Lalinde E. (2023). A new family of luminescent iridium complexes: Synthesis, optical, and cytotoxic studies. Dalton Trans..

[35] Ezquerro C., Sepulveda A.E., Grau-Atienza A., Serrano E., Lalinde E., Berenguer J.R., Garcia-Martinez J. (2017). Organometallic phosphors as building blocks in sol-gel chemistry: Luminescent organometallo-silica materials. J. Mater. Chem. C.

[36] Ezquerro C., López I., Serrano E., Alfaro-Arnedo E., Lalinde Peña E., Larráyoz I., Pichel J.G., García-Martinez J., Berenguer J.R. (2022). Highly Emissive Hybrid Mesoporous Organometallo-Silica Nanoparticles for Bioimaging. Mater. Adv..

[37] Bryden M.A., Millward F., Lee O.S., Cork L., Gather M.C., Steffen A., Zysman-Colman E. (2024). Lessons learnt in photocatalysis—The influence of solvent polarity and the photostability of the photocatalyst. Chem. Sci..

[38] Bruker AXS Inc (2021). APEX4, SAINT, and SADABS.

[39] Sheldrick G.M. (2015). SHELXT—Integrated space-group and crystal-structure determination. Acta Crystallogr. Sect. A Found. Adv..

[40] Sheldrick G.M. (2015). Crystal structure refinement with SHELXL. Acta Crystallogr. Sect. C Struct. Chem..

[41] Farrugia L.J. (1999). WinGX suite for small-molecule single-crystal crystallography. J. Appl. Crystallogr..

[42] Spek A.L. (2003). Single-crystal structure validation with the program PLATON. J. Appl. Crystallogr..

[43] Spek A.L. (2015). PLATON SQUEEZE: A tool for the calculation of the disordered solvent contribution to the calculated structure factors. Acta Crystallogr. Sect. C Struct. Chem..

[44] Frisch M.J., Trucks G.W., Schlegel H.B., Scuseria G.E., Robb M.A., Cheeseman J.R., Scalmani G., Barone V., Petersson G.A., Nakatsuji H. (2016). Gaussian 16. 3.

[45] Becke A.D. (1993). Density-functional thermochemistry. III. The role of exact exchange. J. Chem. Phys..

[46] Grimme S., Antony J., Ehrlich S., Krieg H. (2010). A consistent and accurate ab initio parametrization of density functional dispersion correction (DFT-D) for the 94 elements H-Pu. J. Chem. Phys..

[47] Wadt W.R., Hay P.J. (1985). Ab initio effective core potentials for molecular calculations. Potentials for main group elements Na to Bi. J. Chem. Phys..

[48] Barone V., Cossi M. (1998). Quantum Calculation of Molecular Energies and Energy Gradients in Solution by a Conductor Solvent Model. J. Phys. Chem. A.

[49] O’Boyle N.M., Tenderholt A.L., Langner K.M. (2008). cclib: A library for package-independent computational chemistry algorithms. J. Comput. Chem..

