# Supplementary material for: Sol-Gel Heterogeneization of an Ir(III) Complex for Sustainable Visible-Light Redox Photocatalysis
Source: Molecules. 2025 Apr 9;30(8):1680. doi: 10.3390/molecules30081680 (PMC12029348; doi:10.3390/molecules30081680)
Supplement: Supplementary file 1 [file molecules-30-01680-s001.zip › molecules-3546382-supplementary.pdf]

Supporting Information

Sol-Gel Heterogeneization of an Ir(III) Complex for Sustainable Visible-Light Redox Photocatalysis

Janira Herce, Mónica Martínez-Aguirre, Javier Gómez-Benito, Miguel A. Rodríguez* and Jesús R. Berenguer*

Departamento de Química-Instituto de Investigación en Química (IQUR), Universidad de La Rioja, Madre de Dios 53, Logroño E-26006, Spain. E-mail: jesus.berenguer@unirioja.es; miguelangel.rodriguez@unirioja.es

Table of content

Schemes and NMR spectra for the synthesis of complexes 1 and 2 3

X-ray diffraction data of [Ir(dfbzapy)$_2$(pyraphen)]PF_6·(1·1.5acetone) 5

Solid state MALDI spectra 6

TEM images and electron diffraction pattern 7

Photophysical and electronic characterization 8

Theoretical calculations 10

Photocatalytic study 13

Photostability studies 14

Schemes and NMR spectra for the synthesis of complexes 1 and 2

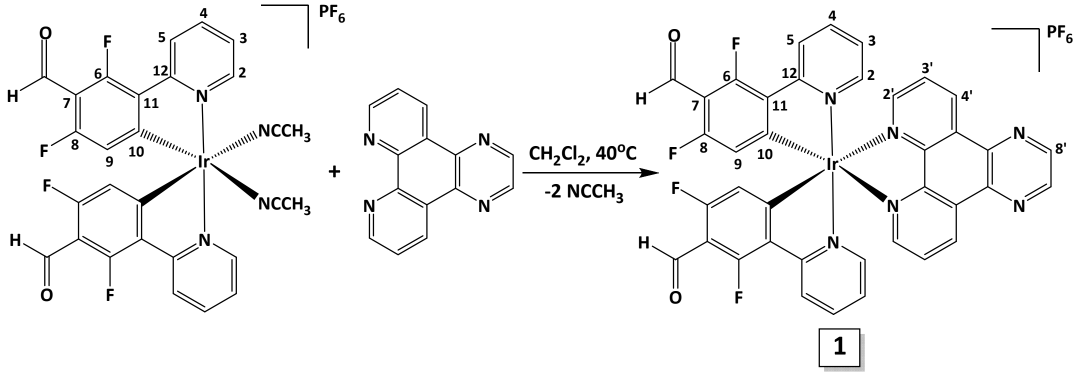

Scheme S1. Synthesis of complex **1**, showing the numbering scheme used in the NMR characterization.

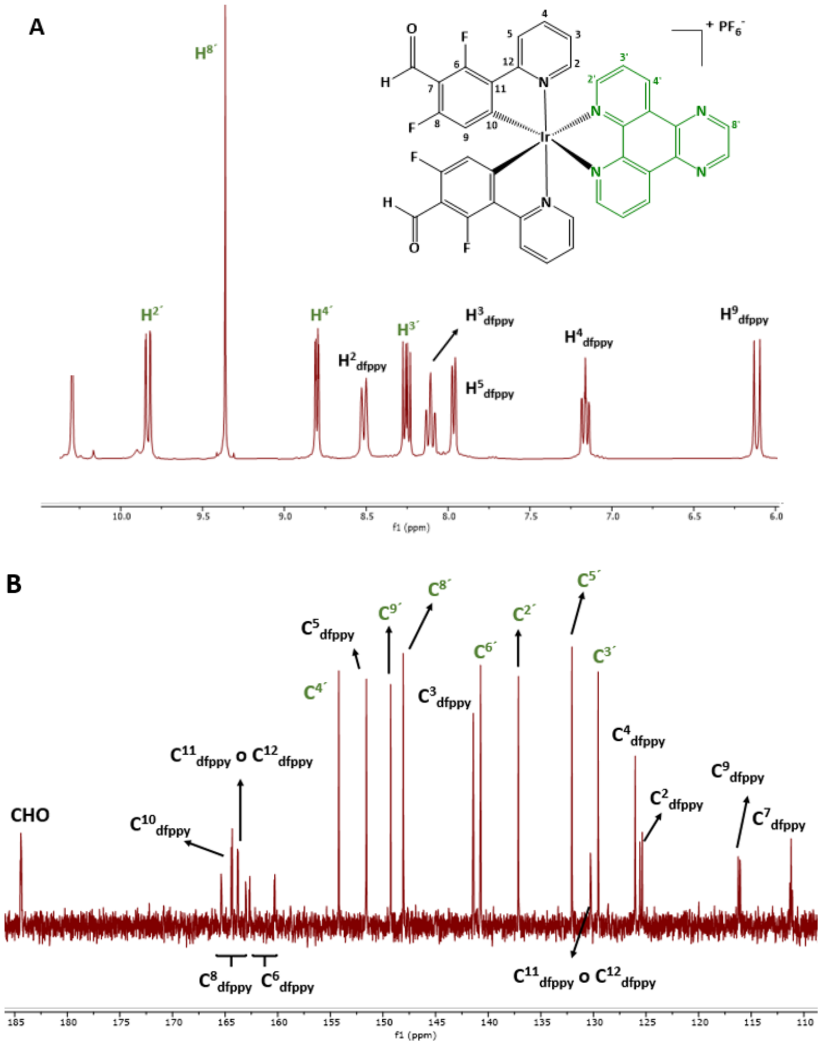

Figure S1. A) ^{1}H and B) $^{13}C\{^{1}H\}$ NMR spectra of complex **1** in acetone-d_6.

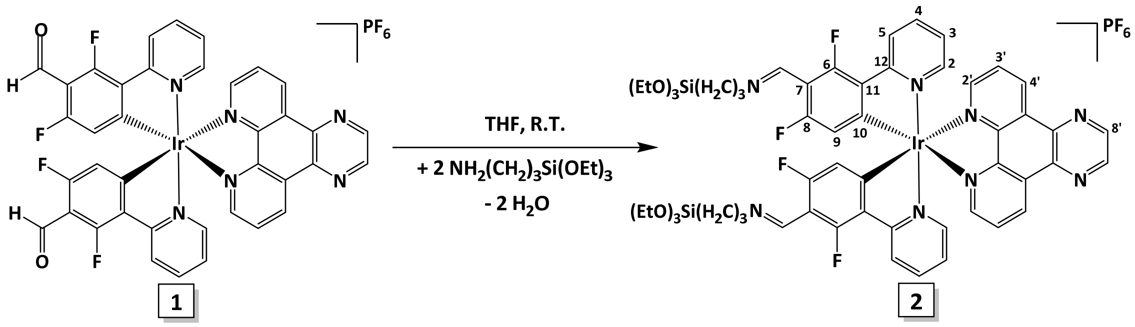

Scheme S2. Synthesis of complex **2**, showing the numbering scheme used in the NMR characterization.

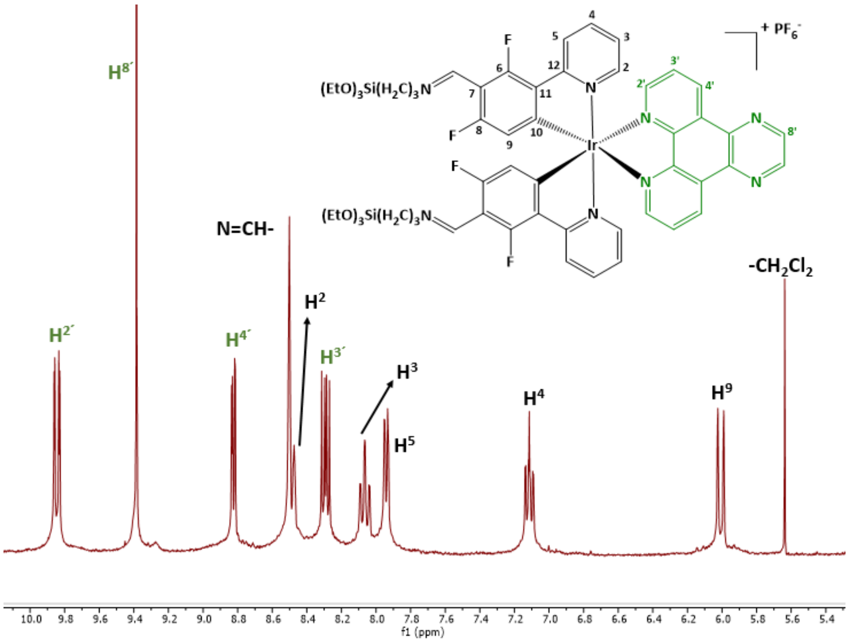

Figure S2. ^1H NMR spectra of complex **2** in acetone-d_6.

X-ray diffraction data of [Ir(dfbzapy)$_2$(pyraphen)]PF_6·(1·1.5acetone)

Table S1. Selected distances (Å) and angles (°) for **1**·1.5$(CH_3)_2CO$.

Ir(1)-C(10)	2.006(4)	Ir(1)-N(1)	2.044(4)
Ir(1)-N(2)	2.141(2)	C(13)-O(1)	1.210(8)
N(1)-Ir(1)-C(10)	80.6(2)	N(1)-Ir(1)-C(10’)	93.8(2)
N(1)-Ir(1)-N(2)	98.5(2)	N(1)-Ir(1)-N(2’)	87.6(2)
N(1)-Ir(1)-N(1’)	172.3(2)	C(10)-Ir(1)-C(10’)	86.2(2)
N(2)-Ir(1)-C(10)	98.2(2)	N(2)-Ir(1)-C(10’)	175.6(2)
N(2)-Ir(1)-N(2’)	77.4(2)	C(7)-C(13)-O(1)	123.6(6)

Table S2. X-ray Crystallographic Data for **1**·1.5$(CH_3)_2CO$.

Empirical formula	$C_{38}H_{20}F_{10}IrN_6O_2P$
Fw	1005.77
T(K)	100(1) K
Crystal system, space group	Monoclinic, C 2/c
a(Å)	11.1918(6)
b(Å)	27.5994(15)
c(Å)	12.8509(7)
α(deg)	90
β(deg)	104.831(2)
γ(deg)	90
Volume (A^3)	3837.2(4)
Z	4
Dcalcd ($Mg{\cdot}m^{-3}$)	1.741
Absorption coefficient (mm^{-1})	3.613
F(000)	1952
θ range for data collection (deg)	2.906 to 28.813
No. of data // restraints // params	4970 // 0 // 263
Goodness-of-fit on F^{2} (a)	1.119
Final R indexes [$I>2\sigma(I)$](a)	R1 = 0.0370, wR_2 = 0.0981
R indexes (all data)(a)	R1 = 0.0515, wR_2 = 0.1013
Largest diff peak and hole ($e{\cdot}Å^{-3}$)	2.261 and -1.700

(a) $R_1 = \Sigma(|F_o| - |F_c|)/\Sigma|F_o|$; $wR_2 = [\Sigma w(F_o^2 - F_c^2)^2/\Sigma wF_o^2]^{1/2}$; goodness of fit = $\{\Sigma[w(F_o^2 - F_c^2)^2]/(N_{obs} - N_{param})\}^{1/2}$; $w = [\sigma^2(F_o) + (g_1P)^2 + g_2P]^{-1}$; $P = [\max(F_o^2;0 + 2F_c^2]/3$.

Solid state MALDI spectra

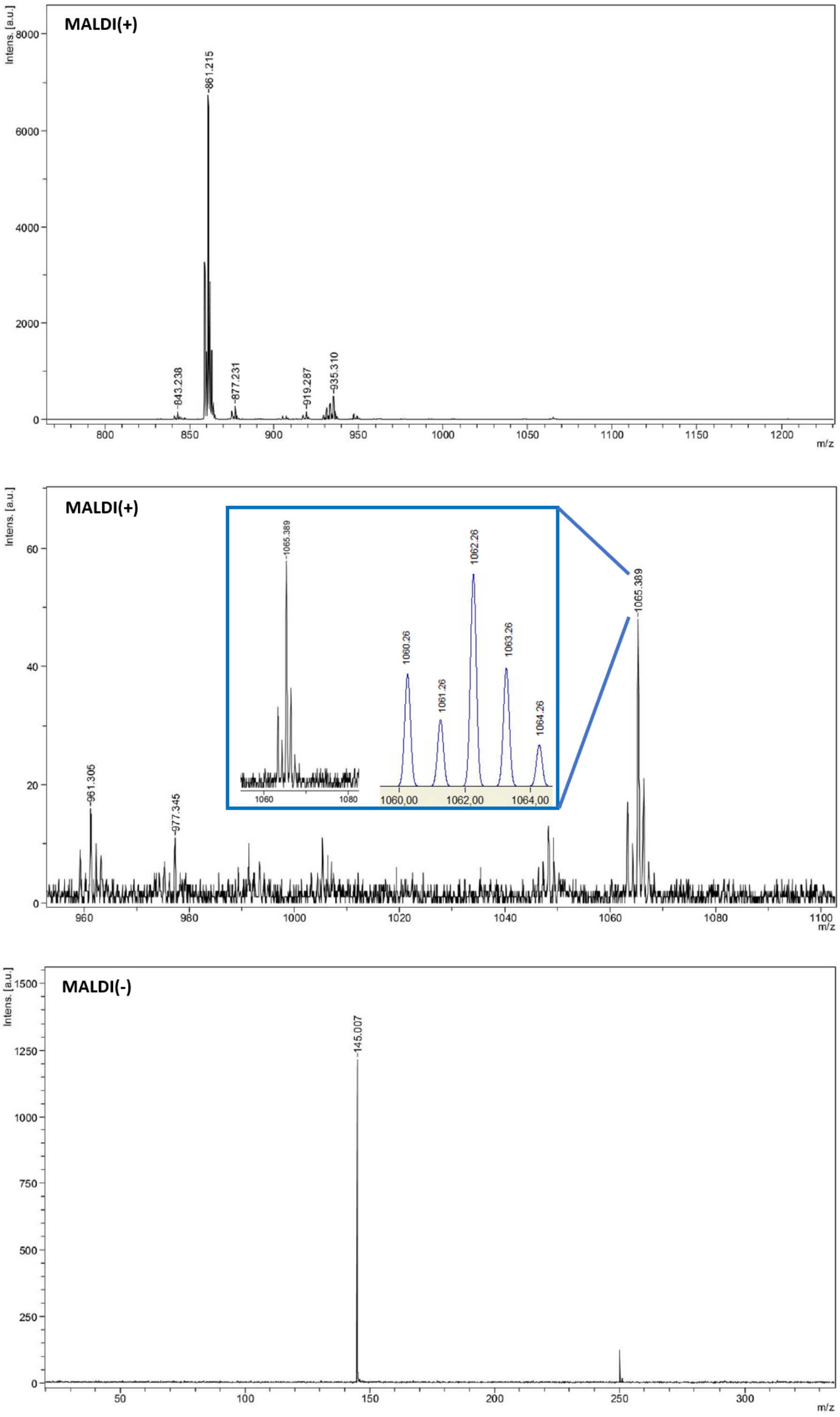

Figure S3. The solid-state MALDI spectra of the self-condensed material **SC-2**, including an expanded view of the peak corresponding to the cationic ionization fragment at $^{m}/_{z}$ [**SC-2** – {$(CH_2)_3Si(OEt)_3$} + 3H]$^+$ (1%), alongside the simulated isotopic distribution (MALDI(+)). The base peak, observed at $^{m}/_{z}$ 861, is attributed to the loss of the pyraphen ligand, yielding the species [**SC-2** – {$(CH_2)_3Si(OEt)_3$} – (pyraphen) + Et + 2H]$^+$ (100%). In the MALDI(-) spectrum, a single signal at $^{m}/_{z}$ 145 is detected, corresponding to the PF_6^- anion.

TEM images and electron diffraction pattern

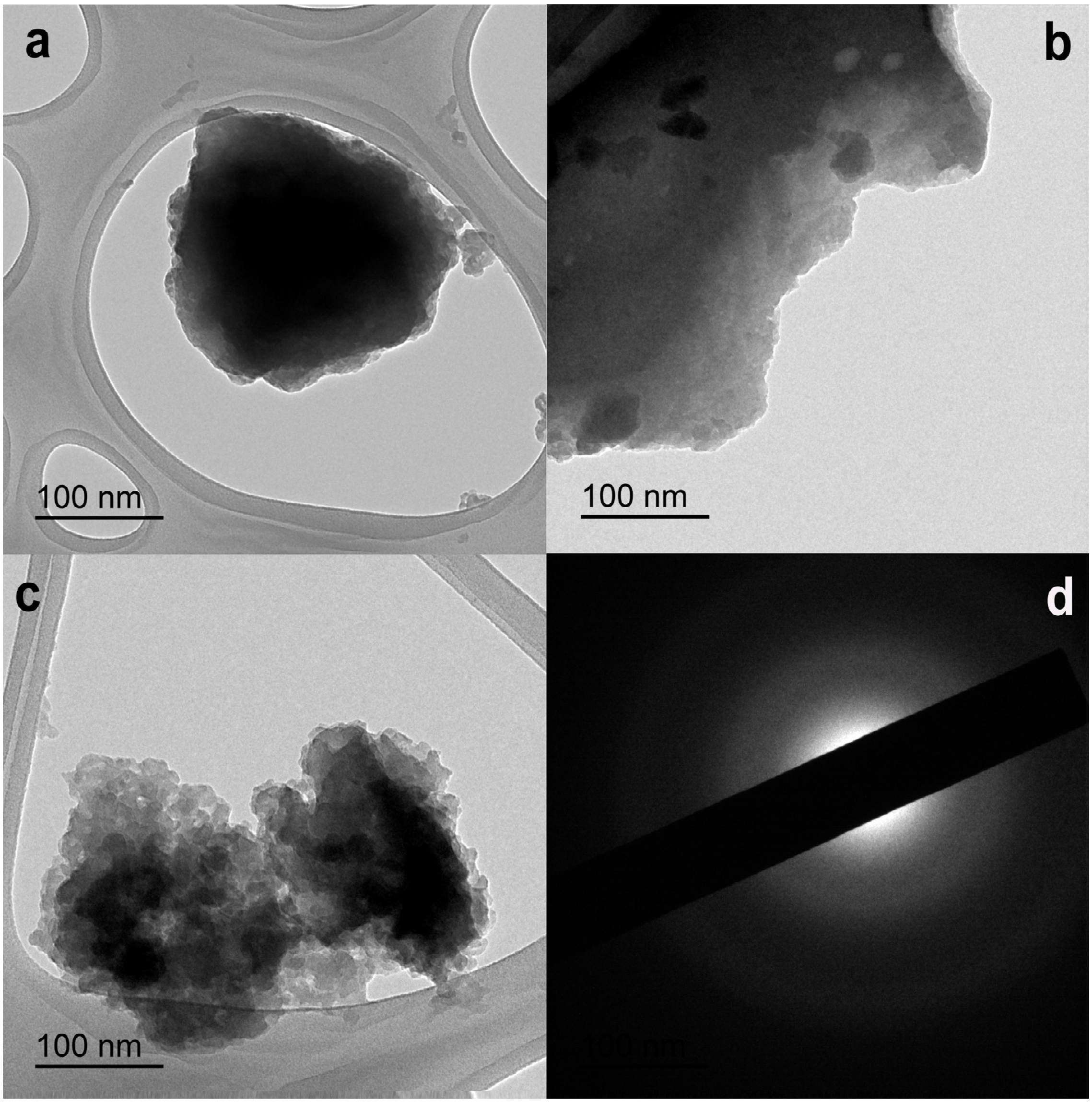

Figure S4. Representative TEM images (a-c) and the electron diffraction pattern (d) of **SC-2**.

Photophysical and electronic characterization

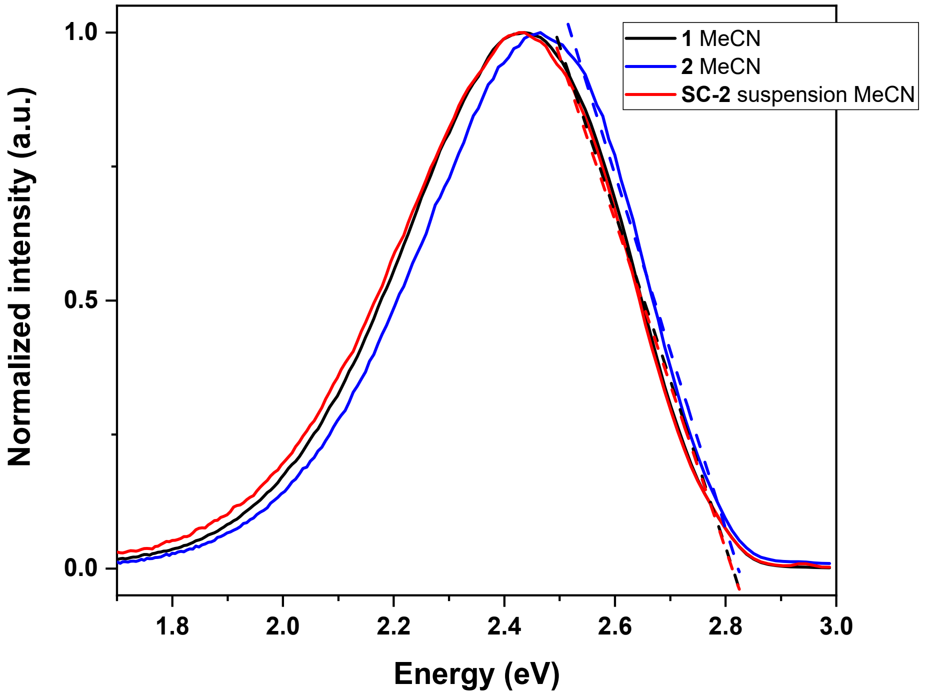

Figure S5. Normalized emission spectra of complex **1** and **2** in solution (5·10^{-4} M) and **SC-2** in suspension (1 mg/mL) of MeCN at room temperature, with the tangential onset used to calculate $E_T(T_1)$.

Table S3. Experimental optical gaps [$E_{0,0}(T_1)$] and $E_T(T_1)$ values of complex **1** and **2** in solution (5·10^{-4} M) and self-condensed material **SC-2** in suspension (1 mg/ml) of MeCN.

Sample	MeCN	
	$E_{0,0}$/eV$^{a)}$	T_1/eV$^{b)}$
**[Ir(dfppy)$_2$(pyraphen)]PF$_6$ (1)**	2.78	2.83
**[Ir(Si-dfppy)$_2$(pyraphen)]PF$_6$ (2)**	2.78	2.84<br>(2.74)
**SC-2**	2.85	2.83

a) $E_{0,0}$ determined from the intersection point between the normalized excitation and emission spectra in MeCN. b) T_1 determined from the tangential onset from the emission spectra in MeCN at room temperature. Values in parentheses indicate the $E_T(T_1)$ values obtained from DFT calculations for complex **2**.

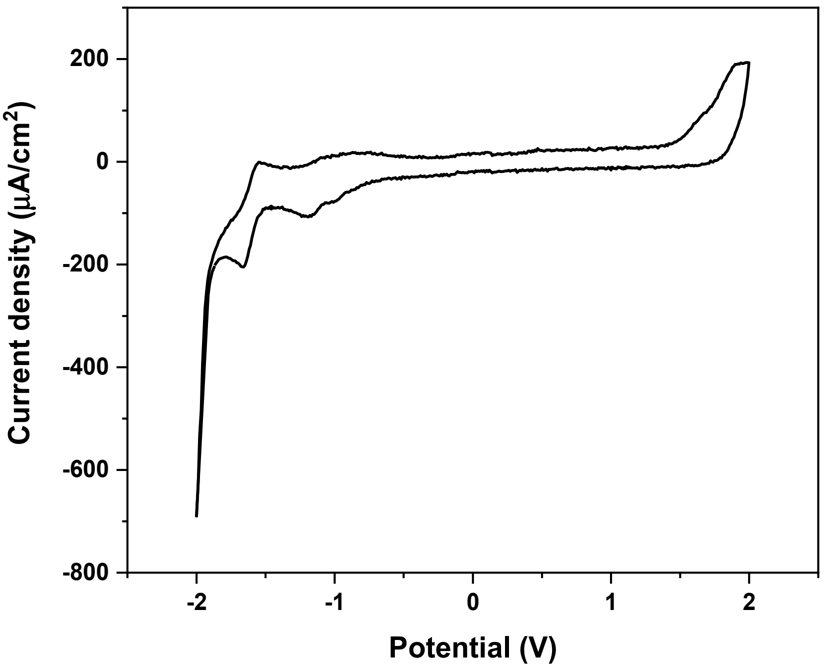

Figure S6. Cyclic Voltammogram obtained for complex **2** in MeCN solution (5 10^{-4} M) at scan rate of 100 mV·S^{-1}.

Table S4. Electrochemical properties and calculated frontiers orbitals for complex **2** in MeCN solution (5·10^{-4} M).

$E_{ox}^{1/2,a)}$/V	$E_{red}^{1/2,a)}$/V	ΔE/V	HOMO/eV[b)]	LUMO/eV[b)]	E^{*}_{ox}/V [c)]	E^{*}_{red}/V [c)]
1.72	-1.61	3.33 (3.35)	-6.08 (-5.98)	-2.75 (-2.63)	-1.06	1.17

a) $E_{ox}^{1/2}$ and $E_{red}^{1/2}$ are the anodic and cathodic potentials. b) The HOMO and LUMO energy level are referenced to 0.44 V of Fc/Fc^{+} couple potential. HOMO/LUMO = -[$E_{ox/red}^{1/2}$-0.44+4.8]. c) E^{*}_{ox} = $E_{ox}^{1/2}$ – $E_{0,0}$ and E^{*}_{red} = $E_{red}^{1/2}$ + $E_{0,0}$. Values in parentheses indicate the values obtained from DFT calculations.

Theoretical calculations

Table S5. Composition (%) of Frontier MOs in the ground state for complex **2** in MeCN.

MO	eV	Ir	pyraphen	dfbzapy (1)	dfbzapy (2)
LUMO+5	-1.34	1	3	47	49
LUMO+4	-1.87	4	1	47	47
LUMO+3	-1.96	5	1	47	47
LUMO+2	-2.37	0	99	0	0
LUMO+1	-2.45	1	98	0	0
LUMO	-2.63	2	98	0	0
HOMO	-5.98	31	1	34	34
HOMO-1	-6.45	3	1	48	49
HOMO-2	-6.65	13	1	43	42
HOMO-3	-6.72	18	5	38	39
HOMO-4	-6.87	57	6	18	18
HOMO-5	-6.91	41	16	22	22

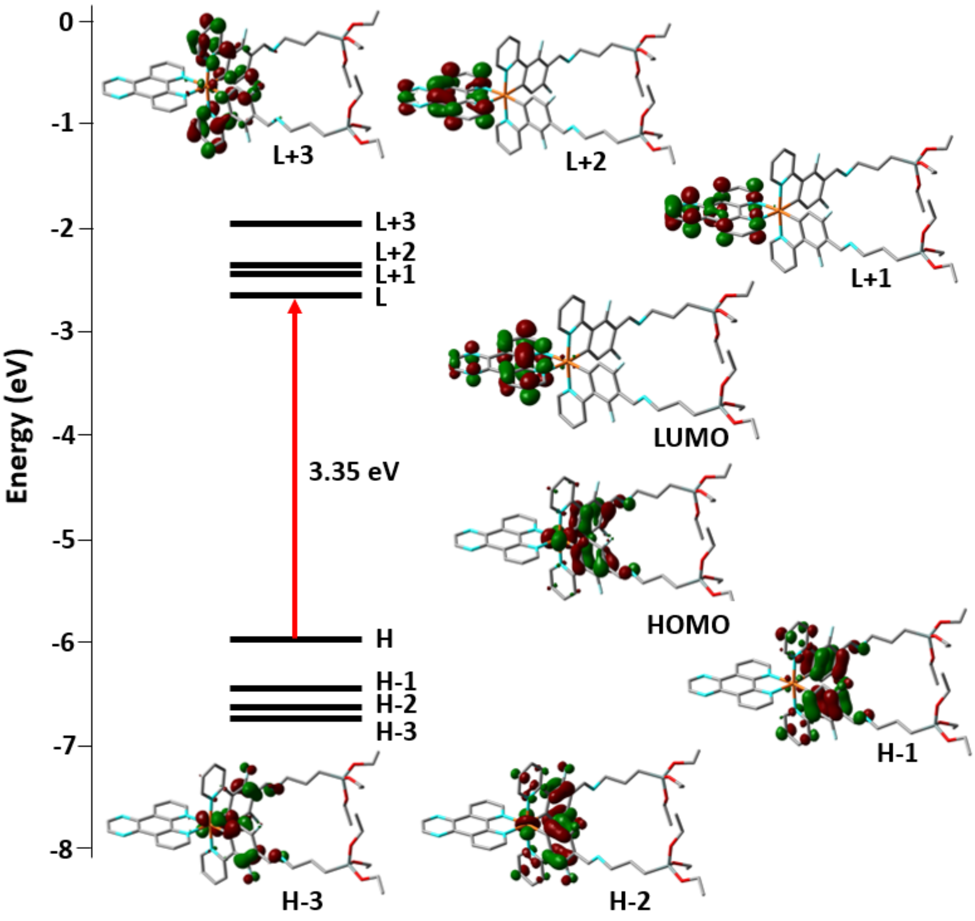

Figure S7. Selected Frontier Molecular Orbitals for complex **2** in acetonitrile, showing the HOMO-LUMO gap.

Table S6. Selected vertical excitation energies singlets (S_0) and the first triplet computed by TDDFT/SCRF with the orbitals involved for **2** in MeCN.

State	λ/nm	f	Transition (% Contribution)	Main Character
T_1	456.45	-	HOMO→LUMO (84%)	ML'CT/ LL'CT
T_2	444.64	-	H-1→L+4 (17%), HOMO→L+3 (47%)	MLCT
T_3	440.39	-	H-1→L+3 (27%), HOMO→L+4 (33%), HOMO→L+7 (11%)	MLCT
S_1	451.82	0.0004	HOMO→LUMO (90%)	ML'CT/ LL'CT
S_2	402.60	0.0005	HOMO→L+2 (98%)	ML'CT/ LL'CT
S_3	402.43	0.0005	HOMO→L+1 (90%)	ML'CT/ LL'CT
S_4	375.71	0.0659	HOMO→L+3 (96%)	IL/MLCT
S_5	371.64	0.0239	H-1→LUMO (88%)	LL'CT
S_6	365.02	0.0001	H-4→LUMO (37%), H-2→LUMO (51%)	ML'CT/ LL'CT
S_7	361.30	0.0081	HOMO→L+4 (89%)	MLCT
S_8	351.21	0.1335	H-5→LUMO (27%), H-3→LUMO (54%)	ML'CT/ LL'CT
S_9	344.38	0.0004	H-4→LUMO (45%), H-2→LUMO (40%)	ML'CT/ LL'CT
S_{10}	343.21	0.001	H-1→L+1 (92%)	LL'CT
S_{11}	341.08	0.0037	H-1→L+2 (89%)	LL'CT
S_{12}	333.34	0.0029	H-9→LUMO (39%), H-9→L+1 (57%)	IL/ML'CT/ LL'CT
S_{34}	296.85	0.4358	H-5→L+4 (11%), H-3→L+4 (29%), HOMO→L+5 (20%), HOMO→L+6 (26%)	ML'CT/ L'LCT
S_{38}	291.41	0.2071	H-4→L+3 (40%), H-3→L+4 (10%), H-2→L+3 (28%)	MLCT/ L'LCT
S_{46}	283.50	0.1003	H-5→L+4 (63%), H-3→L+4 (17%)	MLCT/ L'LCT
S_{47}	283.04	0.4349	H-11→L+1 (27%), HOMO→L+7 (36%)	MLCT/ L'LCT
S_{54}	268.45	0.5339	H-1→L+5 (59%)	LL'CT
S_{56}	266.15	0.1532	H-1→L+7 (45%)	LL'CT
S_{60}	263.30	0.4951	H-12→L+2 (18%), H-1→L+6 (48%)	ML'CT
S_{65}	260.08	0.3929	H-12→LUMO (18%), H-11→L+2 (26%), H-2→L+5 (27%)	MLCT/ LL'CT
S_{67}	258.97	0.2135	H-5→L+5 (11%), H-3→L+5 (24%), H-2→L+7 (30%)	ML'CT/ L'LCT
S_{71}	256.90	0.1295	H-2→L+7 (11%), H-1→L+6 (16%)	MLCT/ LL'CT
S_{72}	256.32	0.2381	H-3→L+7 (12%), H-2→L+6 (19%), H-1→L+8 (28%)	MLCT
S_{76}	254.06	0.3539	H-4→L+5 (13%), H-3→L+7 (24%), H-3→L+8 (22%), H-1→L+7 (11%)	MLCT
S_{80}	252.58	0.2606	H-5→L+6 (10%), H-3→L+5 (15%), H-3→L+6 (32%), H-2→L+8 (15%)	MLCT/ L'LCT

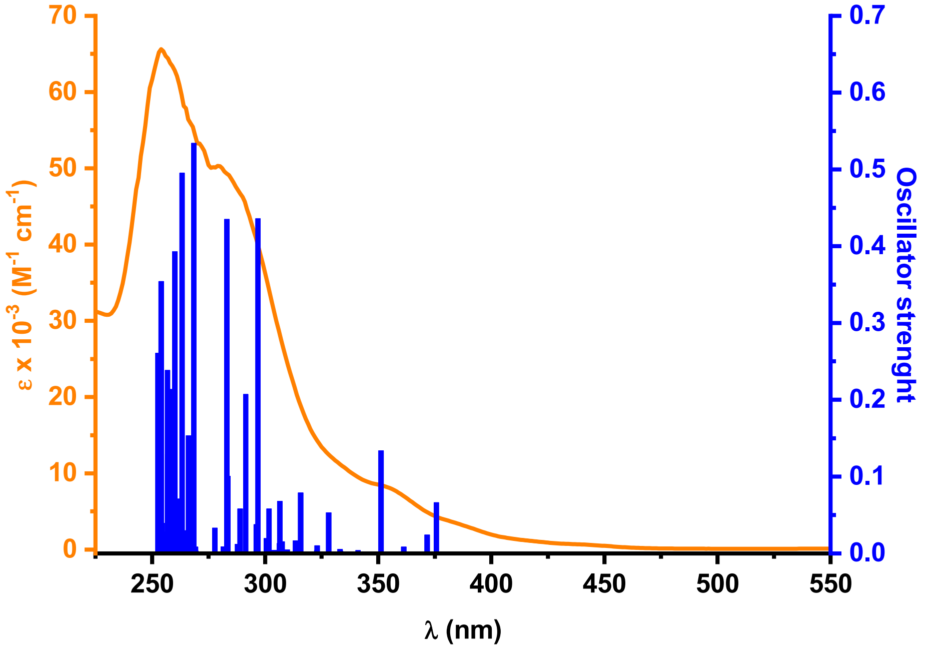

Figure S8. Calculated stick absorption spectra of **2** in MeCN compared with the experimental data.

Table S7. Composition (%) of Frontier MOs in terms of ligands and metals in the first triplet state for **2** in MeCN.

MO	eV	Ir	pyraphen	dfbzapy (1)	dfbzapy (2)
**SOMO**	-3.47	2	98	0	0
**SOMO-1**	-5.23	36	3	31	31

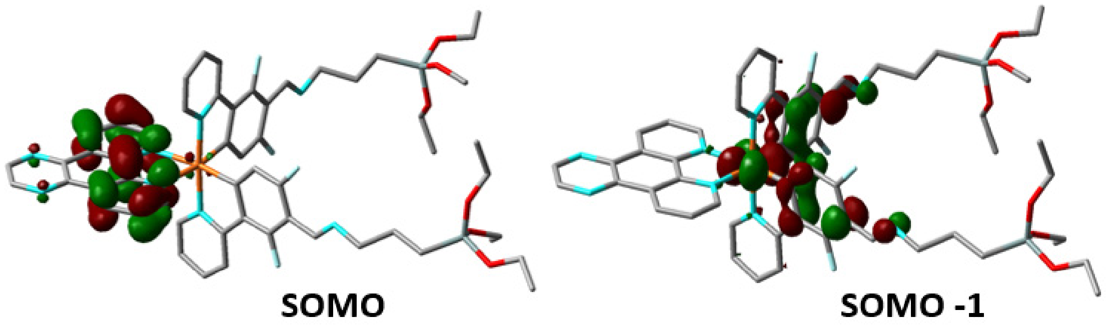

Figure S9. SOMO and SOMO-1 orbitals for the first excited triplet state in complex **2**.

Photocatalytic study

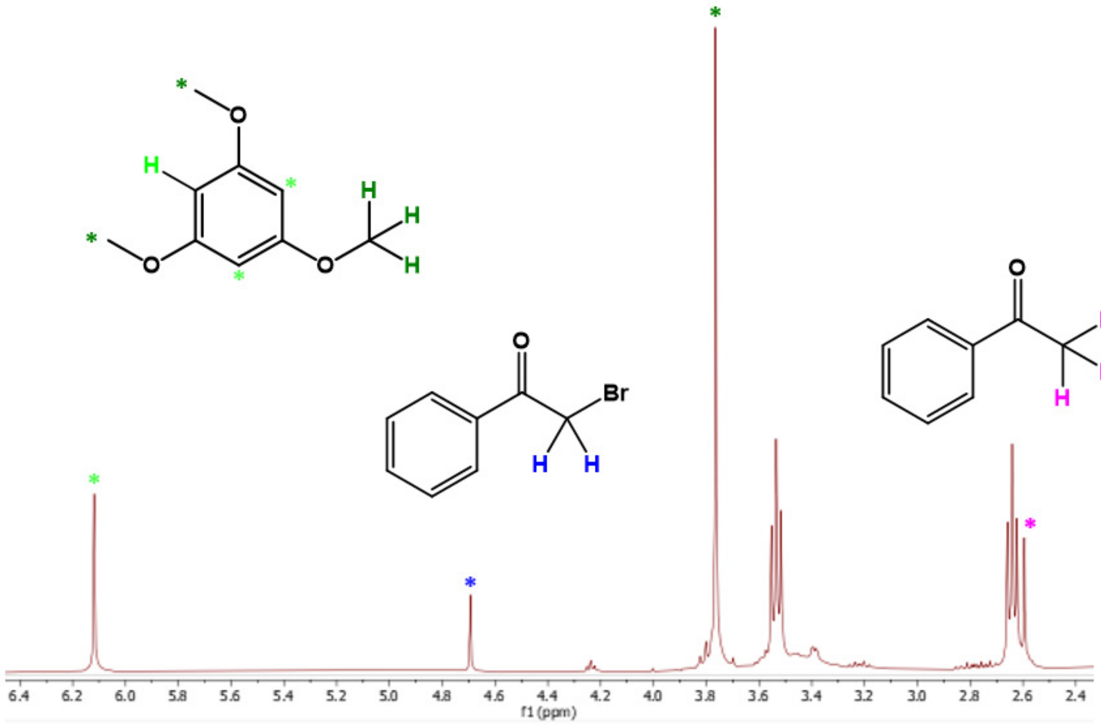

Figure S10. Singlet signals observed in the ^{1}H NMR spectrum were employed to quantify the reaction yield during the photocatalytic dehalogenation of 2-bromoacetophenone.

Photostability studies

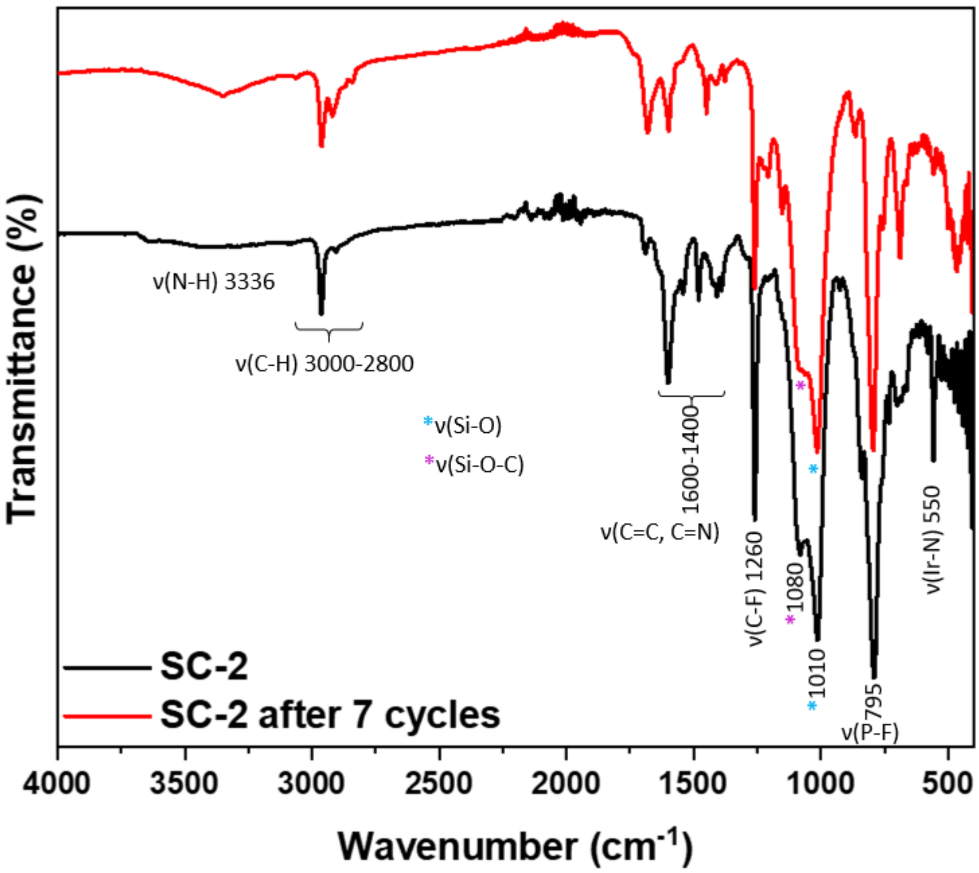

Figure S11. IR spectra of **SC-2** before and after the photocatalytic reaction.

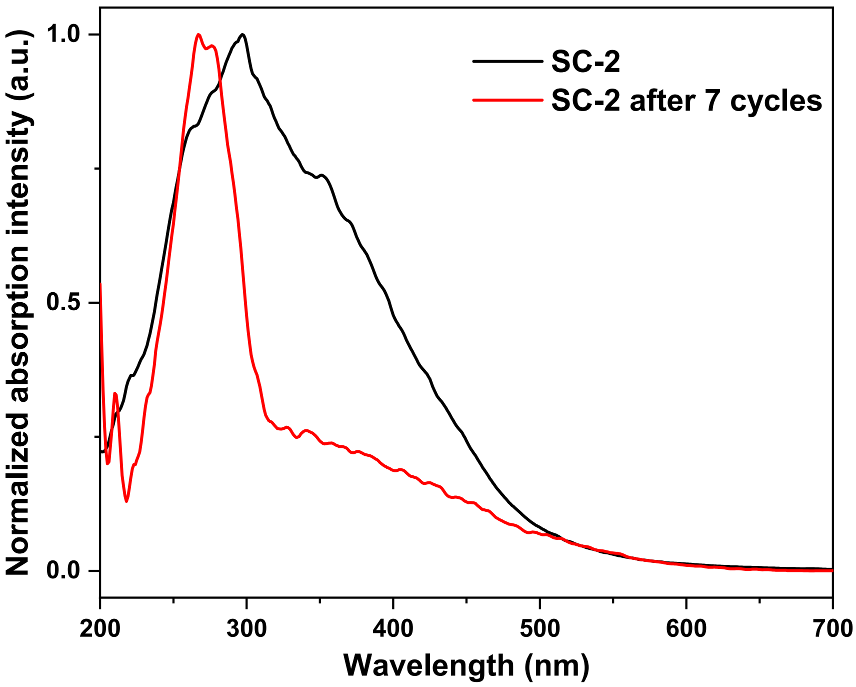

Figure S12. Solid-state absorption spectra of **SC-2** before and after the photocatalytic reaction.

Table S8. Absorption data of material **SC-2** in solid form before and after use in 7 consecutive cycles.

Sample	λ_{abs} / nm
**SC-2 solid**	256, 295, 350, 375, 420, 445
**SC-2 after 7 cycles**	267, 278, 325, 340, 385, 407, 450

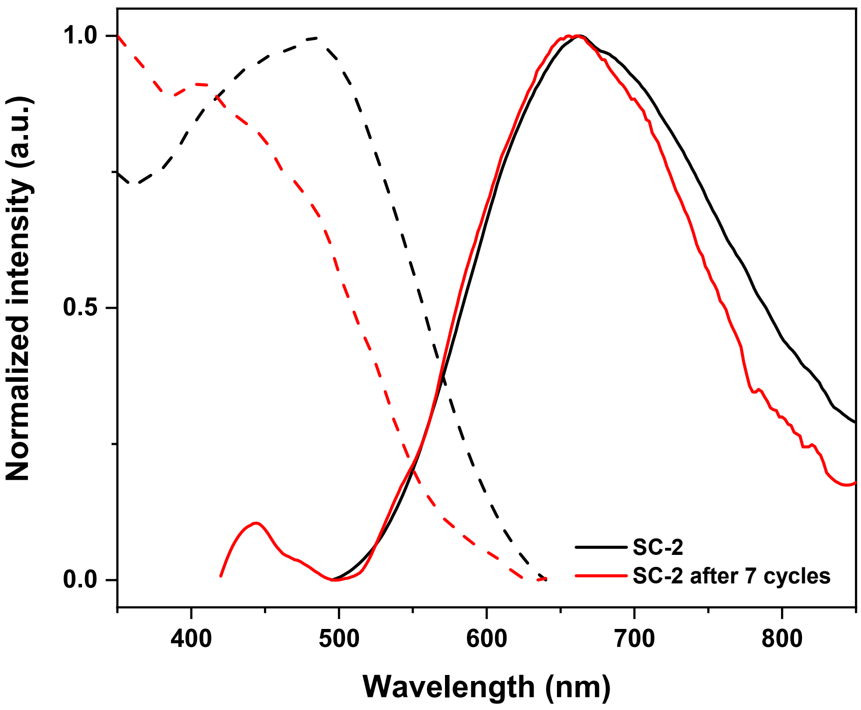

Figure S13. Solid-state excitation and emission spectra of **SC-2** before and after photoctalytic reaction.

Table S9. Photophysical data in solid state of **SC-2** before and after being used in seven cycles of the dehalogenation of 2-bromoacetophenone. All data at Room Temperature.

Sample	λ_{em}/ nm (λ_{exc}/nm)	τ/μs
**SC-2**	660 (475)	10.73
**SC-2** after 7 cycles	660 (400)	13.66
